# Sub-Chronic Effects of Slight PAH- and PCB-Contaminated Mesocosms in *Paracentrotus lividus* Lmk: A Multi-Endpoint Approach and De Novo Transcriptomic

**DOI:** 10.3390/ijms22136674

**Published:** 2021-06-22

**Authors:** Luisa Albarano, Valerio Zupo, Davide Caramiello, Maria Toscanesi, Marco Trifuoggi, Marco Guida, Giovanni Libralato, Maria Costantini

**Affiliations:** 1 Stazione Zoologica Anton Dohrn, Department of Marine Biotechnology, Villa Comunale, 80121 Naples, Italy; luisa.albarano@szn.it (L.A.); giovanni.libralato@unina.it (G.L.); 2 Department of Biology, University of Naples Federico II, Complesso Universitario di Monte Sant’Angelo, Via Cinthia 21, 80126 Naples, Italy; marco.guida@unina.it; 3 Stazione Zoologica Anton Dohrn, Department of Marine Biotechnology, Villa Dohrn, Punta San Pietro, 80077 Naples, Italy; vzupo@szn.it; 4 Stazione Zoologica Anton Dohrn, Department of Research Infrastructures for Marine Biological Resources, Marine Organisms Core Facility, Villa Comunale, 80121 Naples, Italy; davide.caramiello@szn.it; 5 Dipartimento di Scienze Chimiche, Università degli Studi di Napoli Federico II, Complesso Universitario di Monte S. Angelo, Via Cintia, 80126 Naples, Italy; maria.toscanesi@unina.it (M.T.); marco.trifuoggi@unina.it (M.T.)

**Keywords:** de novo transcriptomic, marine sediment, polycyclic aromatic hydrocarbons, polychlorinated biphenyls, sea urchin, short-term effects

## Abstract

Sediment pollution is a major issue in coastal areas, potentially endangering human health and the marine environments. We investigated the short-term sublethal effects of sediments contaminated with polycyclic aromatic hydrocarbons (PAHs) and polychlorinated biphenyls (PCBs) on the sea urchin *Paracentrotus lividus* for two months. Spiking occurred at concentrations below threshold limit values permitted by the law (TLVPAHs = 900 µg/L, TLVPCBs = 8 µg/L, Legislative Italian Decree 173/2016). A multi-endpoint approach was adopted, considering both adults (mortality, bioaccumulation and gonadal index) and embryos (embryotoxicity, genotoxicity and de novo transcriptome assembly). The slight concentrations of PAHs and PCBs added to the mesocosms were observed to readily compartmentalize in adults, resulting below the detection limits just one week after their addition. Reconstructed sediment and seawater, as negative controls, did not affect sea urchins. PAH- and PCB-spiked mesocosms were observed to impair *P. lividus* at various endpoints, including bioaccumulation and embryo development (mainly PAHs) and genotoxicity (PAHs and PCBs). In particular, genotoxicity tests revealed that PAHs and PCBs affected the development of *P. lividus* embryos deriving from exposed adults. Negative effects were also detected by generating a de novo transcriptome assembly and its annotation, as well as by real-time qPCR performed to identify genes differentially expressed in adults exposed to the two contaminants. The effects on sea urchins (both adults and embryos) at background concentrations of PAHs and PCBs below TLV suggest a need for further investigations on the impact of slight concentrations of such contaminants on marine biota.

## 1. Introduction

Sediments are composed of soluble, insoluble (rock and soil particles) and biogenic matter, which can be naturally transported from lands to oceans due to coastal erosion and windblown dust [1]. Sediment represents an essential and dynamic part of marine environments and may accumulate organic and/or inorganic compounds deriving from natural and anthropogenic sources, such as industrial, commercial, agricultural and urban activities [2,3]. Contamination associated with (re-)suspended sediment is a concern for human health, mainly due to its tendency to accumulate in bottom-feeder organisms and biomagnify through marine food webs [4,5]. Worldwide governments are promoting sediment assessment, restoration and valorization as a key compartment of water bodies [6] (i.e., the European Union via the Water Framework (2000/60/EC) (WFD) and the Marine Strategy Framework Directives (2008/56/EC)). Natural (i.e., tides, bioturbation) and artificial (i.e., dredging) perturbative events can remobilize sediment and dissolve the associated contaminants into the water column, including polycyclic aromatic hydrocarbons (PAHs) and polychlorinated biphenyls (PCBs), causing short- and long-term effects on marine organisms [7]. PAHs consist of a large group of widespread organic compounds of high environmental concern, occurring mainly in relation to human activities, such as combustion by-products (i.e., atmospheric deposition) [8] or oil spillage (approximately 15% *w/w* of PAHs); nevertheless, crude oil water-soluble fraction effects (i.e., mixture) are still largely unexplored [9,10].

It has been estimated that direct discharges of PAHs in marine environments can range from <1 µg/L to over 625 µg/L, with concentrations in industrial effluents up to 4.4 mg/L and 170.000 ng/g in sediment (dry weight; [11]). PCBs are a group of anthropogenic compounds, classified as persistent organic pollutants (POPs) by the Stockholm Convention (2001). Less than 1% of PCBs released in the environment volatilize from soil/sediment to the atmosphere, while most of them accumulate into the water column and in aquatic organisms, and they reach up to 4601 ng/g dry weight in the sediment [12].

Besides pollution hotspots (i.e., industrial and commercial sites), marine sediments are generally only slightly contaminated by PAHs and PCBs, with concentrations below nationally and internationally set threshold limit values (TLVs) (TLV_PAHs_ = 900 µg/L, TLVPCBs = 8 µg/L; Legislative Italian Decree 173/2016), but our knowledge about the potential side effects of this background contamination on human health and the environment is still insufficient.

This research investigated the potential negative effects of PAH (to simulate post- combustion products) and PCB slightly contaminated sediment on the sea urchin *Paracentrotus lividus* Lamark. Ad hoc experimental mesocosms [13] were set up to expose adult sea urchins to a reconstructed marine habitat (i.e., sediment and water) purposely spiked with PAHs and PCBs [14,15]. We tested two hypotheses proposing that the effects of slightly polluted sediment could result in the following: (i) morphological changes in the development of sea urchin embryos, deriving from adults exposed to these contaminants and (ii) variation in the expression level of genes involved in stress response, skeletogenesis, detoxification and development/differentiation. Specifically, sea urchin endpoints included adult’s mortality; gonadal index; sensitivity of embryos (up to pluteus stage) generated from the exposed organisms; contaminant accumulation in adult thecae and spines, gonads and intestine; genotoxicity; and de novo transcriptome assembly.

## 2. Results and Discussion

### 2.1. Sediment Grain Size and Water Features and Spiking Levels 

The sediment showed a typical sandy profile. Sandy fraction represented 99.9%, where the coarse sand (0.5 mm–1 mm, representing 41.1%) was the dominant component (Supplementary Figure S1). The fine and medium sands (from 0.25 mm to 0.5 mm) were 2.1% and 15.7%, respectively, whereas the mud fraction represented a small percentage (about 0.1%). During two months of exposure to PAH- and PCB-contaminated sediments, the physical and chemical values of the seawater in the mesocosm were almost constant. PAHs and PCBs detected in all sediment and water samples from all mesocosms were below the relative detection limit values (see Supplementary Table S1–S8 for more details) considering all investigation times (t0, t1 and tf).

### 2.2. Effects of Contaminated Sediment on Adult Growth, Gonadal Index and Sea Urchin Development

None of the conditions imposed in the negative controls (W and W + SED) negatively affected sea urchins, suggesting that all the subsequently observed effects could be attributed to the treatments. After the exposure period (two months), a mortality rate of 1% was detected in all experimental conditions (W, W + SED, W + SED + PAHs and W + SED + PCBs), revealing good health conditions of the sea urchins after two months of exposure (Supplementary Figure S2). After two months of exposure, no significant differences in growth rates were found between adults exposed to PAHs and PCBs as compared to organisms collected in the field at the beginning of the experiment (*p* > 0.05), similar to GI values (*p* value > 0.05; Supplementary Table S9). 

**Figure 1:**
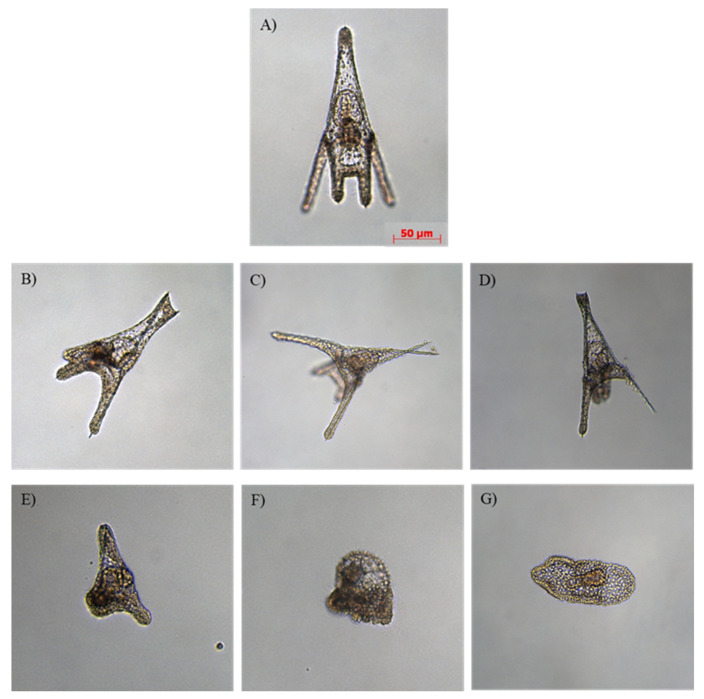
Examples of malformations observed in (**B**–**E**) *p. lividus* plutei deriving from adults exposed to PAHs and PCBs and in (**F**,**G**) embryos still at the gastrula stage deriving from adults exposed to PCBs in comparison with (**A**) control embryos deriving from adults reared in a tank with sediment without contaminants. (**B**) poorly-formed apex; (**C**) crossed at the apex with wider aperture of the arms; (**D**) degraded arms; (**E**) delayed and abnormal body; (**F**,**G**) malformed gastrulae.

After gamete collections, three important endpoints of sea urchin embryonic development were detected: (i) fertilization success; (ii) first mitotic division; and (iii) the pluteus stage, occurring at 48 hpf. Exposure to both contaminants, PAHs and PCBs, did not show significant effects on the percentages of fertilization success and first mitotic cleavage with respect to the controls (in tanks with seawater (W) and in tanks with seawater + sediment (W + SED) without contaminants; *p* > 0.05). Observation of the embryos at the pluteus stage revealed that PAH and PCB treatments induced malformations, mainly affecting arms, spicules, apices and the entire body shape as compared to control embryos (Figure 1).

**Figure 2:**
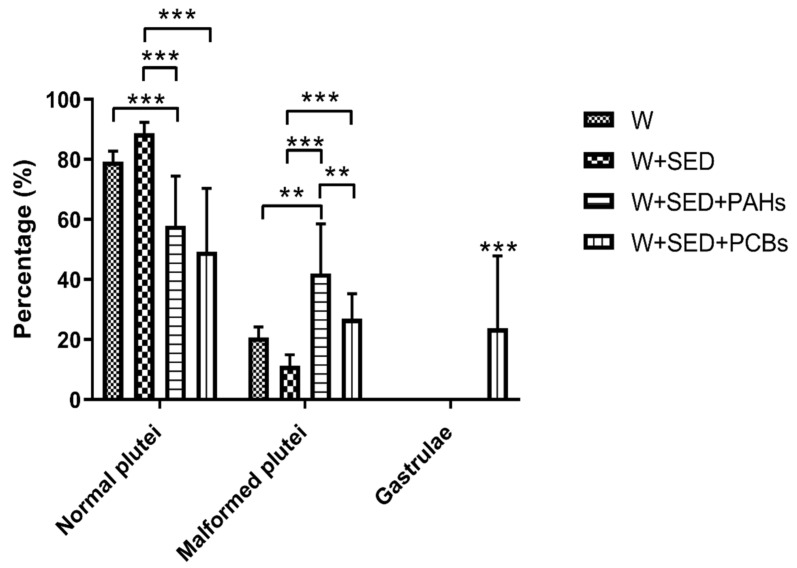
Percentage of normal plutei and malformed embryos at the pluteus and gastrula stages from sea urchins, deriving from adult sea urchins exposed to sediment contaminated with PAHs (water + sediment + PAHs) and PCBs (water + sediment + PCBs) and in control conditions represented by adults reared in control tanks (water and water + sediment). Data are reported as mean ± standard deviation one-way ANOVA by Holm–Sidak test (** *p* < 0.01, *** *p* < 0.001).

In particular, at the pluteus stage, an increase in malformed embryos was observed in larvae deriving from sea urchins exposed to contaminated sediments with respect to the controls, represented by water and water + sediment without contaminants (Figure 2). 

PAHs induced an increased percentage of malformed embryos (about 42%) with respect to control water + sediment (about 10%, *p* < 0.001). The exposure to PCBs generated approximately 27% (*p* < 0.001) of malformed plutei and developmental delays, with some embryos still at the gastrula stage (about 24%; *p* < 0.001), which were also malformed.

These results, valid for the above-mentioned doses applied to *P. lividus*, demonstrated that PAHs are more harmful than PCBs, being supported also by chemical analyses of the contaminant’s bioaccumulation.

After two months of exposure, the bioaccumulation of PAHs and PCBs was also detected in three sea urchin tissues: thecae (including spines), gonads and guts. Chemical results showed that (i) 12.4 µg/kg of PAHs (including acenaphthylene, acenaphthene, fluorene, anthracene, phenanthrene, 9-methylanthracene and benzo[a]anthracene) were accumulated in the theca, including the spine (Table 1); (ii) 16.3 µg/kg of total PAHs (including acenaphthylene, acenaphthene, fluorine, anthracene, phenanthrene, fluoranthene, pyrene and benzo[a]anthracene) were accumulated in the gonads; and (iii) no PAH accumulation was found in the guts. The target body compartments in sea urchins were the body wall and the spines when individuals were exposed to contaminated water and the guts when they were exposed to contaminated foods [16].

However, the accumulation in these marine organisms was more efficient when exposed via water than via the food. No detectable events of PCB bioaccumulation were observed in the analyzed tissues (Supplementary Table S10). PCB bioaccumulation data on marine organisms are scarce, impeding an effective assessment of their toxicity. Zeng et al. [17,18] studied the uptake patterns of PCB congeners in the sea urchin *Lytechinus pictus*. More than 66 days are necessary for some congeners to attain steady state concentration in *L. pictus* gonad, whereas 28–42 days are required [19] in such marine organisms as bivalves, polychaetes and amphipods. Evidence of toxicity with changes in total or gonad weight was only detected at 647 mg/g. Studies on fish indicated that embryos and developing larvae were negatively affected by PCBs at 0.12 mg/g–12 mg/g [20,21]. Monosson et al. [20] observed that PCB effects were due to the congener 3,3′,4,4′ tetrachlorobiphenyl, which has a greater toxicity than that of the congeners’ mixture [16], and exposed adult *P. lividus* to 14C-labelled PCB#153 via seawater and food, observing that the bioaccumulation efficiency was similar in the body wall, spines, gut and gonads.

### 2.3. Transcriptomic Assembly and Differentially Expressed Genes in Plutei from Adults Exposed to PAHs and PCBs (RNA-seq)

Another interesting result was the large-scale genomic information herein reported, which greatly improved the few molecular tools available for the sea urchin *P. lividus*, despite its importance as a marine model organism. For this reason, the de novo transcriptome obtained in this work represents a promising tool to identify new *P. lividus* genes, which can be considered general biomarkers placed in motion from the sea urchin to deal with environmental pollution.

All the results obtained by RNA sequencing are summarized below.

BLASTx top-hit species distribution of matches for all the transcriptomes with known sequences indicated (Supplementary Figure S3) that the majority of *P. lividus* contigs (reads) showed the highest similarity with *Strongylocentrotus purpuratus* (BLAST hits = 1000). The other most represented species included *Apostichopus japonicas* (BLAST hits 50) and *Acanthaster planci* (BLAST hits 45). All alignments were carried out setting the E-value thresholds at a value of ≤1 e^−5^.

To perform the RNA-seq assembly de novo, Trinity was used [22]. We obtained the trinity assembly with the statistics reported: Counts of transcripts: Total “trinity genes”: 216864, Total “trinity transcripts”: 611356, Percent GC: 38.26%. Then, we performed a differentially expression analysis in Trinity, selecting Deseq2 R package [23], and we obtained the genes differentially expressed with respect to the several conditions (less than 3000 genes and 8000 isoforms). Of the isoforms differentially expressed, we performed a BLASTx alignment with respect to the nucleotide non-redundant database in NCBIi, using OmicsBox (version1.2.4) [24]. Differentially expressed genes were identified between the three conditions: embryos at the pluteus stage spawned by adults exposed for two months to sediment contaminated with (i) PAHs or (ii) PCBs, with comparisons made with (iii) those exposed in tanks with sediment without contaminants as the control, including three biological replicates for each treatment.

The score plot showed that the replicates for the controls were very similar, with a clear separation from the treated samples, suggesting a greater number of down- and up-regulated genes in the treated samples compared to that of the controls (Supplementary Figure S4).

As reported in Supplementary Table S11, (i) 1898 genes were differentially expressed (DE) genes with a false discovery rate (FDR) of ≤ 0.05, of which 993 genes were up-regulated (FC ≥ 1.5) and 965 were down-regulated (FC ≤ 1.5) in plutei deriving from adult *P. lividus* exposed to sediment contaminated with PAHs (indicated as Treated_1); (ii) 2396 genes were DE with a false discovery rate (FDR) of ≤ 0.05, of which 1079 genes were up-regulated (FC ≥ 1.5) and 1317 were down-regulated (FC ≤ 1.5) in plutei deriving from adult *P. lividus* exposed to sediment contaminated with PCBs (indicated as Treated_2) compared to the control; (iii) 1356 genes were DE with a false discovery rate (FDR) of ≤ 0.05, of which 755 genes were up-regulated (FC ≥ 1.5) and 601 were down-regulated (FC ≤ 1.5), considering Treated_1 compared to Treated_2. After the annotation, (i) 488 genes were found up-regulated (with a FC range between 1.6 and 99) and 271 genes down-regulated (with a FC range between 1.7 and 95) for Treated_1 vs. Control, and, of these, some genes showed very high values of fold changes, such as the four up-regulated genes (RNA-directed DNA polymerase from mobile element jockey-like, calmodulin-like protein 4, arylsulfatase A and fibropellin-1-like isoform X6) and the three down-regulated genes (beta-1,3-galactosyltransferase 1-like, isocitrate dehydrogenase (NADP) cytoplasmic isoform X2 and fibrillin-1-like); (ii) 311 genes were found up-regulated (with a FC range between 1.7 and 95) and 420 genes down-regulated (with a FC range between 1.6 and 95) for Treated_2 vs. Control, and, of these, some genes showed very high values of fold changes, such as the three up-regulated genes (dnaJ homolog subfamily B member 13, rho guanine nucleotide exchange and factor 39ATP-dependent RNA helicase DHX8-like) and the two down-regulated genes (betaine-aldehyde dehydrogenase and serine/threonine-protein kinase TNNI3K); (iii) 177 genes were found up-regulated (with a FC range between 1.9 and 90) and 239 genes down-regulated (with a FC range between 1.5 and 98) for Treated_1 vs. Treated_2, and, of these, some genes showed very high values of fold changes, such as the three up-regulated genes (actin-related protein 2/3 complex subunit 3, acyl-CoA dehydrogenase and arylsulfatase I) and the two down-regulated genes (cyclin-dependent kinase 11B and glucose-6-phosphate 1-dehydrogenase isoform X1).

**Figure 3:**
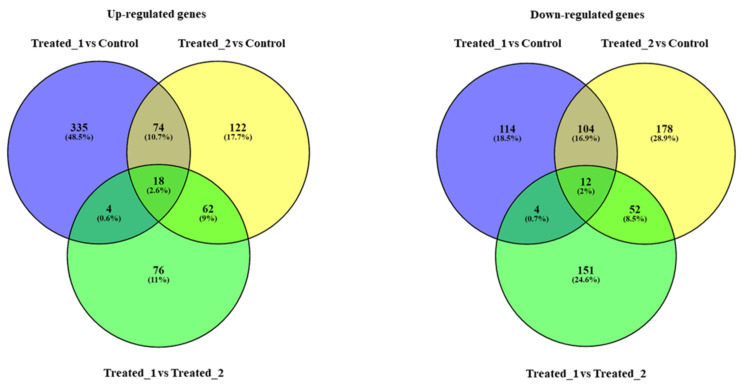
Venn diagrams considering up-regulated genes and down-regulated genes, comparing the groups “Treated_1 (plutei deriving from adults exposed for two months to sediment contaminated with PAHs) versus Control (plutei from adults sea urchin *P. lividus* reared for two months in tanks with sediment without contaminants)”, “Treated_2 (plutei deriving from adults exposed for two months to sediment contaminated with PCBSs) versus Control” and “Treated_1 versus Treated_2”. PAHs (Treated_1) and PCBs (Treated_2) induced an increase in the expression of 335 (48.5%) and 122 (17.7%) genes, respectively, compared to the Control; they also induced the down-regulation of 114 (18.5%) and 178 (28.9%) genes, respectively. The two contaminants had several common targets (see also Supplementary Tables S12 and S13 for the names of the common genes): (i) for up-regulated genes, 74 common genes (10.7%) comparing the groups “Treated_1 versus Control” and “Treated_2 versus Control”; 18 common genes (2.6%) comparing the groups “Treated_1 versus Control”, “Treated_2 versus Control” and “Treated_1 versus Treated_2”; 4 common genes (0.6%) comparing “Treated_1 versus Control” and “Treated_1 versus Treated_2”; 62 common genes (9.0%) comparing “Treated_2 versus Control” and “Treated_1 versus Treated_2”. (ii) for down-regulated genes, 104 common genes (16.9%) comparing the groups “Treated_1 versus Control” and “Treated_2 versus Control”; 12 common genes (2.0%) comparing the groups “Treated_1 versus Control”, “Treated_2 versus Control” and “Treated_1 versus Treated_2”; 4 common genes (0.7%) comparing “Treated_1 versus Control” and “Treated_1 versus Treated_2”; 52 common genes (8.5%) comparing “Treated_2 versus Control” and “Treated_1 versus Treated_2”.

This large-scale genomic information represents a significant finding, being the first molecular attempt to define PAH and PCB effects on sea urchin *P. lividus* by molecular approaches. PAHs and PCBs targeted different genes and had several common targets, as shown in the Venn diagrams considering up-regulated genes and down-regulated genes, comparing the groups “Treated_1 (plutei deriving from adults exposed for two months to sediment contaminated with PAHs) versus Control (plutei from adults sea urchin *P. lividus* reared for two months in tanks with sediment without contaminants)”, “Treated_2 (plutei deriving from adults exposed for two months to sediment contaminated with PCBSs) versus Control” and “Treated_1 versus Treated_2”(Figure 3 and Supplementary Tables S12 and S13).

**Figure 4:**
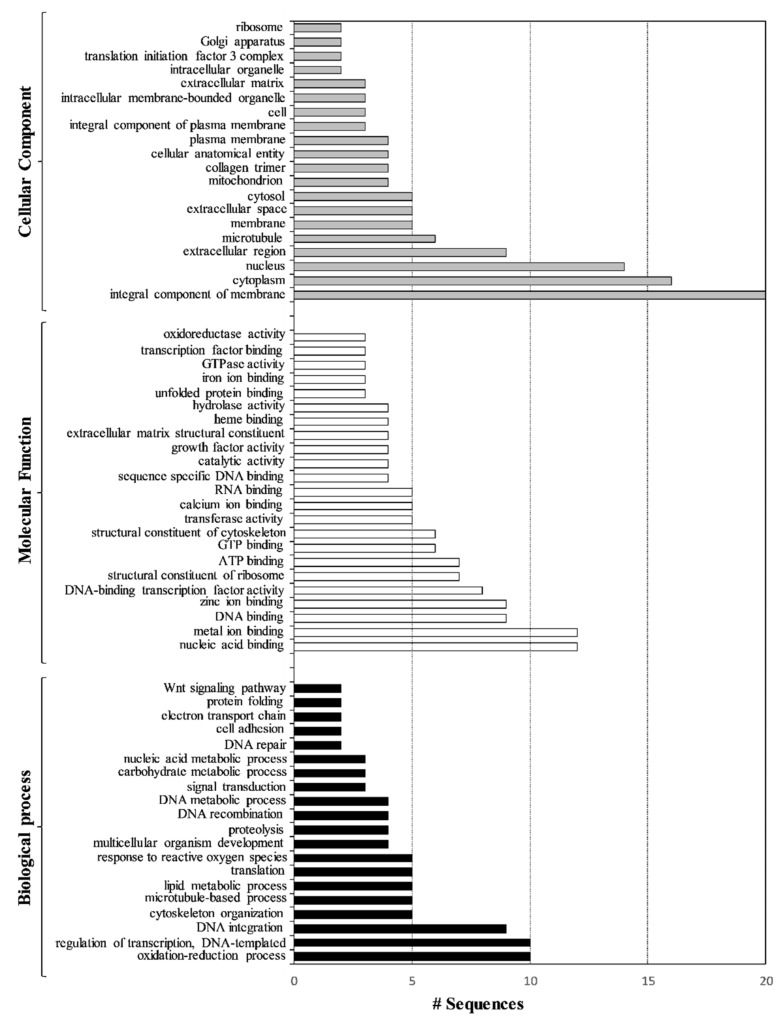
Overrepresented GO terms of sea urchin plutei after artificial contaminated experiments with PAHs and PCBs in the three major functional categories: Biological Process (black bars), Molecular Function (white bars) and Cellular Component (grey bars), which include all the differentially expressed genes (both up- and down-regulated).

Transcriptomic results indicate that PAHs and PCBs affected genes differently, mainly increasing their gene expressions, supporting those differences observed at the morphological level. In fact, the highest percentage of malformed plutei caused by exposure to PAHs can be linked to the up-regulation of the majority of the studied genes. An example was represented by nodal and nectin genes (data also confirmed by real-time qPCR experiments, see below). The nectin gene is involved in cellular adhesion [25,26], whereas nodal gene controls the left–right asymmetry in the sea urchins, regulating the expression level of the *BMP2* gene [27,28,29,30]. The exposure to PCBs caused not only the up-regulation of the nodal gene, but also the up-regulation of the frizzled gene and the down-regulation of the *PLC* gene. The function of the frizzled gene is similar to that of the nodal gene. Binding to the Wnt6, this receptor is responsible for endoderm specification [31,32]. Instead, the *PLC* gene is involved in egg activation in the events immediately following fertilization and during embryo development in sea urchins [33,34,35]. Its down-regulation can be one of the causes of the delay effect shown after PCB treatment. Transcriptome is generally dynamic, and it is a good indicator of the cell’s state. In addition, in this case, the ease of genome-wide profiling made the transcriptome analysis an integral part of understanding the biological processes affected by PAHs and PCBs. In fact, to identify the pathways in which the genes affected by these two contaminants were involved, a Gene Ontology (GO) term enrichment analysis was performed using DE genes (Figure 4).

Seventy-seven GO terms were enriched, including 20 in “Biological Process” followed by 23 in “Molecular Function” and 24 in “Cellular Component” (*p* < 0.05). Over-represented GO categories included the oxidation–reduction process, regulation of transcription, DNA integration, cytoskeleton organization, nucleic acid binding, metal ion binding, DNA binding, zinc ion binding and DNA-binding transcription factor activity. Moreover, these genes are integral components of the membrane and were mainly localized in the cytoplasm, nucleus, extracellular region and microtubule.

### 2.4. Effects of PAHs and PCBs on Gene Expression by Real-Time qPCR

The expression levels of 62 genes [36,37,38,39,40] involved in different physiological processes were followed by a real-time qPCR (reported in Supplementary Figure S5). These genes were previously selected in [36,38,39,41], and their expression levels were studied in response to natural toxins produced by marine diatoms. We proposed these genes as possible biomarkers to detect the consequences of the exposure of marine invertebrates to different environmental pollutants [38]. In particular, these genes were defined as a part of the defensome, which was placed in motion by the sea urchin to protect themselves from environmental toxicants [42].

**Figure 5:**
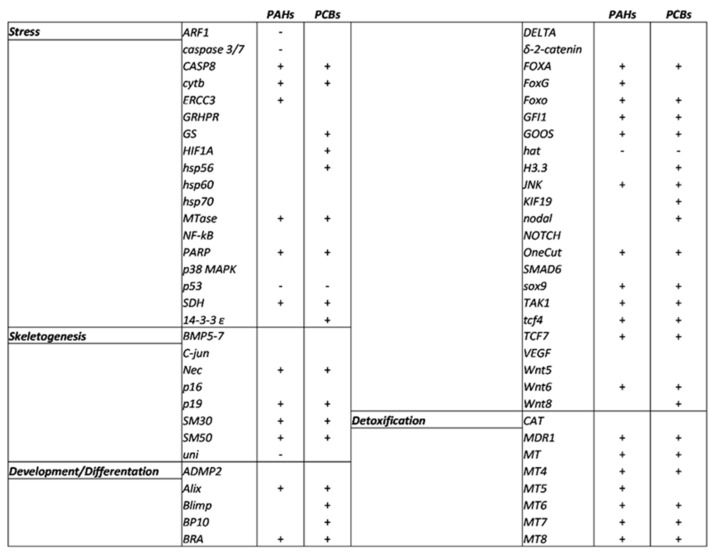
Schematic overview of *P. lividus* genes affected by artificial contaminated sediment with PAHs and PCBs under analysis. + = up-regulated gene; - = down-regulated gene.

At the pluteus stage at 48 hpf (Figure 5, for the numerical values see also Supplementary Table S14), PAHs and PCBs had several common targets.

#### 2.4.1. Stress Genes

Eighteen genes were analyzed, and all were targeted by PAHs and PCBs with the exception of *GRHPR*, *hsp60*, *hsp70*, *NF-kB* and *p38MAPK*. Both contaminants, PAHs and PCBs, increased the expression levels of five genes (*CASP8*, *cytb*, *MTase*, *PARP1* and *SDH*) and decreased that of p53. Moreover, treatment with PAHs also down-regulated *ARF1* and *caspase 3/7* and up-regulated *ERCC3*, whereas the exposure to PCBs up-regulated *GS*, *HIF1A*, *hsp56* and *14-3-3 ε*.

#### 2.4.2. Genes Involved in Development/Differentiation

Among the 28 genes analyzed, only 7 genes (*ADMP2*, *Delta, δ-2-catenin*, *Notch*, *Smad6*, *VEGF* and *Wnt5*) were not targeted by PAHs and PCBs. Common molecular targets for the two contaminants were *Alix*, *Bra*, *FOXA*, *FOXO, GFI1*, *Goosecoid*, *JNK*, *OneCut*, *sox9*, *TAK1*, *tcf4*, and *tcf7*, which were up-regulated, and HAT was the only down-regulated gene. Moreover, *FOXG* was up-regulated only after PAH treatment, whereas PCBs also up-regulated *Blimp*, *BP10*, *H3.3*, *KIF19*, *nodal* and *Wnt8*. PAHs affect axial development and patterning in sea urchin *Lytechinus anemesis* embryos by disrupting the regulation of beta-catenin, a crucial transcriptional co-activator of specific target genes in the Wnt/wg signaling pathway [43].

#### 2.4.3. Skeletogenic Genes

Among the eight genes analyzed, only three genes (BMP5-7, C-jun and p16) were not targeted by the two contaminants. Both PAHs and PCBs increased the expression levels of Nec, p19, SM30 and SM50. Furthermore, PAHs decreased the expression level of the uni gene. The effects of the variations of expression of these genes directly affect the formation of the skeleton of sea urchin embryos. These data were supported by the genes identified in the transcriptomic analysis that belong to biological processes, such as cytoskeleton organization and its structural constituent and the microtubule-based process (see GO terms in Figure 5).

#### 2.4.4. Genes Involved in Detoxification

All eight genes analyzed were targeted by the contaminants with the only exception being the *CAT* gene. *MDR1*, *MT*, *MT4*, *MT6*, *MT7* and *MT8* represented common targets for PAHs and PCBs and were able to increase their expression levels. In addition, the *MT5* gene was only up-regulated by PAHs. PAHs targeted 36 genes and PCBs 40 genes, 31 of which were common molecular targets between them. Genes involved in the detoxification process were also detected in the GO term analysis (see Figure 5).

PAHs and PCBs mainly up-regulated the targeted genes (as in the case of transcriptomic results; Supplementary Figure S5 and Supplementary Table S14) involved in skeletogenesis, developmental/differentiation and detoxification processes, supporting the morphological findings, which revealed that the majority of embryonic malformations affected the skeleton and the developmental plan (Supplementary Figure S6).

To the best of our knowledge, no studies to date have been performed to investigate the effects of PAHs and PCBs on sea urchin *P. lividus* by molecular approaches, with the only exception being Ruocco et al. [44], where the effects of highly contaminated sediments from the site of national interest Bagnoli-Coroglio (Tyrrhenian Sea, western Mediterranean) were detected. Suzuki et al. [45] reported on the effects of benz[a]anthracene and 4-OHBaA on the sea urchin *H. pulcherrimus* plutei, showing that the expression of mRNAs (spicule matrix protein and transcription factors) in the 4-OHBaA-treated embryos was also more strongly inhibited. These results were very similar to those found in our experiments, because *P. lividus* embryos after PAH treatment showed spicule malformation, and the expression levels of the SM30 and SM50 genes were also affected.

These molecular results, completed and deepened by de novo transcriptome, well supported our morphological findings, revealing that the majority of affected genes by both PAHs and PCBs were involved in skeleton formation, in the developmental plan and differentiation of sea urchin, as well as the observed malformations of the embryos, as reported in the GO term analysis (see Figure 4). The up-regulation of these genes identified by real-time qPCR experiments, as well as the up-regulation of genes identified in the de novo transcriptome, lead to the morphological effects detected in embryos deriving from adults exposed to these two contaminants.

## 3. Materials and Methods

### 3.1. Experimental Design and Mesocosms

Our experimental design included four scenarios: (i) negative control—seawater (W) (check filtered seawater background quality); (ii) negative control—not spiked sediment in mesocosm filled with W (W + SED) (check reconstructed sediment background quality); (iii) sediment spiked with PAHs (192 µg/L, nominal) (W + SED + PAHs); and (iv) sediment spiked with PCBs (0.15 µg/L, nominal) (W + SED + PCBs). All experiments were carried out in triplicates.

Each of the 12 testing mesocosms, located at the Stazione Zoologica Anton Dohrn, was characterized by an independent and closed seawater recirculation system (Supplementary Figure S6).

A pump (Micra 400 L/h, SICCE, Italy) promoted seawater circulation from the filtration compartment (containing porous ceramic filters, synthetic sponge and Perlon wool) to the other compartment containing the sediment. Each tank (50 × 36 × 48 cm) was filled with 55 L of natural seawater pre-filtered through a 200-micron mesh sock filter, collected from the Gulf of Naples and treated with zeolite and activated carbon for one week to remove most pollutants prior to chemical analyses (see below; Supplementary Tables S15 and S16). The seawater volume was kept constant during the experiment. It was checked daily and, if necessary, topped up with distilled water. The artificial sediment (10 L) present in each mesocosm was produced by mixing 36.5% of quartz sand (0 mm–3 mm, G. Build, s.r.l.) and 62.5% of coarse sand (grain size 0.4–0.8, Arena Silex, Manufacturas Gre, S.A.) and 1% calcium carbonate) [46]. Before spiking and prior to adding sea urchins, mesocosms were aged for one week. Spiking occurred by adding contaminants directly to the mesocosm water column (i.e., simulating a discharge event). One week after spiking, organisms were added to the mesocosms: seven females and three males for each tank. To evaluate the compartmentalization of PAHs and PCBs in sediment and seawater, their concentrations were evaluated: (i) before the addition of contaminants (t0), (ii) after the addition of contaminants (t1) and (iii) at the end (tf) of the experiment. PAH spiking (acenaphthene (ACE), acenaphthylene (ACY), anthracene (ANT), benzo(a)anthracene (BaA), chrysene (CHR), fluoranthene (FLT), fluorine (FLR), phenanthrene (PHE), pyrene (PYR)) required the addition (in each 55 L mesocosm) of 330 µL of a solution prepared by weighing 30 mg of each PAH in 10 mL of acetone/n-hexane (1:1 *v/v*; the nominal concentration of water stock solution is 35.3 g/L). PCB spiking required the addition (in each mesocosm) of 82 µL of a certified standard solution of 100 µg PCB/mL (Ultra Scientifc, Italy). We did not carry out control experiments with PAH diluents (acetone/n-hexane) due to the insignificant volume added (as compared to mesocosm’s volume) and their high volatility.

### 3.2. Grain Size of Sediment

After one week of aging, 50 mL samples were collected from each tank and treated with 10% H_2_O_2_ and distilled water (2:8) for 48 h at room temperature in order to remove salts and organic matter. After drying (24 h at 105 °C), sediment fractions were mechanically separated with multiple vibrating sieves (Ro-Tap Particle Separator, Giuliani, HAVER & BOECKER Oelde Germany) with a 63 µm mesh to distinguish between sandy and silt–clay fractions [47,48]. Each fraction was weighted separately. Gain size data were analyzed with GradiStat software (version 8.0, [49]) and expressed as a percentage of the total dry weight.

### 3.3. Physico-Chemical Analyses

Temperature, dissolved oxygen, redox potential, salinity and pH were checked three times a week (Supplementary Figures S7 and S8). Temperature and dissolved oxygen were detected by a multiparameter probe (YSI 85, Ohio, US); redox (REDuction-Oxidation) state (270 mV) and pH were evaluated using WTW 197-S (SenTix^®^ 41, Göteborg-Sweden) electrodes (7.8–8.1); salinity was measured by a refractometer (Sper Scientific, Scottsdale, Arizona) and fixed with distilled water when its value exceeded 38 ± 1 PSU. The analysis of nutrients included the detection of nitrites (NO_2_^-^), nitrates (NO_3_^-^), phosphates (PO_4_^3-^) and ammonia (NH_3_^-^), using a colorimetric test (HACH Odyssey DR/2500 spectrophotometer, HACH Company Loveland, Colorado, United States) (Supplementary Figures S9 and S10).

For PAHs and PCBs analyses, seawater samples were extracted by a solid-phase extraction (SPE): 1.0 l of water was filtered and preconcentrated on a C18 disk (ENVI, -18 DSK SPE Disk, diam. 47 mm). The analytes were eluted with a solution of 1:1 dichloromethane and n-hexane. The determination of PAHs and PCBs in the sediment was performed by considering 5 g of dry sediment extracted with acetone/n-hexane 1:1 *v/v* (10 mL), using an ultrasonic disruptor (Brason, US). The extract was concentrated to 1 mL in Multivap under nitrogen flow (Multivap, LabTech, Italy). A total of 10 µL of a 1 mg/L solution of internal standard (mixture of deuterated PAHs) was added to the extract and injected to a gas chromatography–mass spectrometry (GC-MS) (MS-TQ8030-Shimadzu, Japan). The limits of detection (LOD) and quantification (LOQ) were calculated, and the average values for the seawater samples were 0.02 μg/L and 0.05 μg/L for PCBs and 0.004 μg/L and 0.01 μg/L for PAHs, respectively. For sediment samples, LOD and LOQ values were 0.03 μg/kg and 0.1 μg/kg for PCB and 0.16 μg/kg and 0.1 μg/kg for PAHs, respectively. Data quality was ensured by certified reference materials (ERM-CA100 (European Commission) for PAHs and QC1033 (Supelco) for PCBs). The recovery percentage was 70%–110% for PAHs and 65%–120% for PCBs [50,51].

For the determination of PAHs and PCBs in sea urchin tissues (thecae, spines, gonads and guts), approximately 3 g of tissues were homogenated and placed in an automatic extractor, under reflux, at 80 °C for 2 h with a 2 M KOH solution in methanol. After extracting with 20 mL of cyclohexane three times, the extract was purified on sodium sulphate, dried in a rotary evaporator and recovered with a 1 mL mixture of hexane/acetone (1:1 *v/v*). The extract was analyzed by GC–MS. The limit of quantification (LOQ) was of 0.4 μg/kg w.w. and 2 μg/kg w.w. for PAHs and PCBs, respectively. The average recoveries of PAHs and PCBs were >70% [52].

### 3.4. Sea Urchin Collection and Exposure, Gamete Collection for Morphological and Molecular Analysis by Real-Time qPCR

Methods for sea urchin collection (according to Italian laws (DPR 1639/68, 09/19/1980 confirmed on 01/10/2000) and the conditions of their exposure in the mesocosms are reported in Ruocco et al. [44]. Animals were fed *Ulva rigida* according to Ruocco et al. [53].

After two months of exposure, sea urchins were collected and their gametes were obtained. Fertilization, embryonic growth until the pluteus stage (48 hpf) and morphological observations were carried out according to Romano et al. [37]. In particular, the percentage of embryos still at the gastrula stage, as well as normal and malformed plutei, were determined 48 h post-fertilization by counting at least 200 embryos for each sample under light microscopy (Zeiss Axiovert 135TV). Pictures were taken using a Zeiss Axiocam connected to the microscope.

Gonadal indices (GIs) were initially evaluated on gonads from five adult sea urchins (t0) (representing the starting point), and evaluations were repeated on five specimens after two months of exposure to PAHs and PCBs as well as in control sediments (W + SED). Animals were weighed, sacrificed and dissected; gonads were weighted for the GI determined (where GI indicates gonadal wet weight (g)/sea urchin wet weight (g) x 100 according to [54]).

Collection of embryos at the pluteus stage (about 5000 sea urchin plutei) for real-time qPCR was performed according to Ruocco et al. [44]. After total RNA extraction was performed using Aurum™ Total RNA Mini kit (BioRad), its amount and integrity were estimated according to Ruocco et al. [55]. Real-time qPCR experimental protocols as reported in Ruocco et al. [56] and Ruocco et al. [57] were followed (Supplementary Figure S4 reported all the analyzed genes). In particular, about 1 μg of RNA was used for cDNAs synthesis by iScript™ cDNA Synthesis kit (Bio-Rad, Milan, Italy), following the manufacturer’s instructions. The expression of each gene was analyzed and normalized against the housekeeping genes *Ubiquitin* and *18S* rRNA, using REST software (Relative Expression Software Tool, Weihenstephan, Germany) based on the Pfaffl method. Relative expression ratios greater than ±1.5 were considered significant. Each real-time qPCR plate was repeated at least twice.

### 3.5. De novo Transcriptome Assembly and Data Analysis

The sequencing was carried out in Genomix4Life S.r.l. (Baronissi, Salerno, Italy) using Illumina Truseq mRNA stranded 2 × 150—NextSeq500. De novo transcriptome assembly and annotation of 9 samples (1–3 triplicates for the control condition, embryos at the pluteus stage deriving from adult sea urchin reared in the mesocosm in tanks with seawater plus sediment (W + SED) without contaminants, indicated with “Control”; 4–6 triplicates for embryos at the pluteus stage deriving from adult sea urchins exposed to PAHs, indicated as “Treated_1”; and 7–9 triplicates for embryos at the pluteus stage deriving from adult sea urchins exposed to PCBs, indicated as “Treated_2”) were carried out to discover differentially expressed genes between the two treatments and to perform functional analysis (Supplementary Table S17).

RNA sequencing was performed in paired-end mode. Fastq underwent quality control using the FastQC tool [1]. The tool Trinity (Trinity Release v2.10.0; [22]) was used to perform transcriptome assembly. Expression analysis was performed by RSEM (version 1.1.21) using default parameters, and expression values were converted to FPKM (fragments per kilobase of exon per million fragments mapped; Roberts et al. 2011). DESeq2 [23] was used to perform the normalization matrix and differentially expressed genes of all samples were considered. OmicsBox (version1.2.4) uses the Basic Local Alignment Search Tool (BLAST) to find sequences similar to the query set in FASTA format. The Gene Ontology (GO) terms were assigned based on annotation with an E-value of 10-5. The full dataset of raw data is deposited in the Sequence Read Archive (SRA database; available at https://www.ncbi.nlm.nih.gov/sra; accession number: SUB6701449; accessed on 15 February 2021).

### 3.6. Statistical Analyses

Morphological data were reported as means ± standard deviations (SD). These data were analyzed by the Shapiro–Wilk normality test and F-test. The statistical significance between groups was performed by one-way ANOVA followed by the Holm–Sidak test (GraphPad Prism version 8 for Windows, GraphPad Software, La Jolla, California, USA, www.graphpad.com, accessed on 15 February 2021) for multiple comparisons, indicating ** *p* < 0.01, *** *p* < 0.001. Statistical differences of GI values between t0 and after two months were evaluated by the Mann–Whitney U test (GraphPad Prism version 8 for Windows, GraphPad Software, La Jolla, California, USA, www.graphpad.com, accessed on 15 February 2021). *P* values > 0.05 were considered not significant.

## 4. Conclusions

We investigated for the first time the subchronic effects on *P. lividus* of slight PAH and PCB contamination in mesocosms (sediment and water) considering a multi-endpoint approach. Generally, the attention is focused on sediment hot spots (i.e., highly spiked sediment from industrial and commercial areas) with long-term historical pollution (i.e., black samples). We decided to refocus on the so-called “blank” samples with very low concentrations of PAHs and PCBs below national and international threshold limit values (TLVPAHs = 900 µg/L, TLVPCBs = 8 µg/L, Legislative Italian Decree 173/2016). Our reconstructed spiked mesocosms always presented PAH and PCB levels below the respective detection limit values (LODs) for both sediment and water samples, meaning that they soon compartmentalized (in less than one week) between sediment, water, biota, air and mesocosm surfaces. Nevertheless, significant biological effects were detected ranging from bioaccumulation and embryotoxicity (PAHs) to the up- and down-regulations of genes (PAHs and PCBs). Variation of gene expression is directly translated at the morphological level in the malformations observed in the embryos, leading to the identification of genes responsible for those defects. However, de novo assembly is a necessary step to assess differential gene expression and also provides an important resource for researchers working with this sea urchin species. In fact, the transcriptional changes detected in this study are corollary, and, in the future, functional studies will need to clearly establish that these genes can be considered as universal biomarkers involved in the response to contaminants in the marine environment.

Finally, the results evidenced that the combination of morphological and molecular approaches can efficiently support a deeper understanding of how marine species can react to the widespread background sediment contamination levels.

## Figures and Tables

**Table 1:** Quantity (µg/kg) of PAHs detected in thecae (including spines), gonads and gut from adult sea urchin *P. lividus* in two experimental conditions after two months: W/W + SED and W + SED + PAHs. The values higher than the threshold values are reported in red. PAH total values are also reported.

	Thecae + Spines (µg/kg)			Gonads (µg/kg)			Intestine (µg/kg)	
	W + SED + PAHs	W	W + SED	W + SED + PAHs	W	W + SED	W + SED + PAHs	W + SED
Naphthalene	<0.4	<0.4	<0.4	<0.4	<0.4	<0.4	<2	<2
Acenaphthylene	**1.0**	<0.4	<0.4	**2.2**	<0.4	<0.4	<2	<2
Acenaphthene	0.5	<0.4	<0.4	**0.7**	<0.4	<0.4	<2	<2
Fluorene	**5.8**	<0.4	<0.4	**1.1**	<0.4	<0.4	<2	<2
Anthracene	**1.1**	<0.4	<0.4	**3.2**	<0.4	<0.4	<2	<2
Phenanthrene	**1.2**	<0.4	<0.4	**0.8**	<0.4	<0.4	<2	<2
9 metilAntracene	**2.2**	<0.4	<0.4	<0.4	<0.4	<0.4	<2	<2
Fluoranthene	<0.4	<0.4	<0.4	**3.0**	<0.4	<0.4	<2	<2
Pyrene	<0.4	<0.4	<0.4	**4.4**	<0.4	<0.4	<2	<2
Benzo(a)Antracene	**0.7**	<0.4	<0.4	**0.9**	<0.4	<0.4	<2	<2
Benzo(b)Fluorantene	<0.4	<0.4	<0.4	<0.4	<0.4	<0.4	<2	<2
Benzo[k]fluoranthene	<0.4	<0.4	<0.4	<0.4	<0.4	<0.4	<2	<2
Benzo(e)Pirene	<0.4	<0.4	<0.4	<0.4	<0.4	<0.4	<2	<2
Benzo[a]pyrene	<0.4	<0.4	<0.4	<0.4	<0.4	<0.4	<2	<2
Indeno[1,2,3-cd]pyrene	<0.4	<0.4	<0.4	<0.4	<0.4	<0.4	<2	<2
Dibenz[a,h]anthracene	<0.4	<0.4	<0.4	<0.4	<0.4	<0.4	<2	<2
Benzo[ghi]perylene	<0.4	<0.4	<0.4	<0.4	<0.4	<0.4	<2	<2
Coronene	<0.5	<0.4	<0.4	<0.4	<0.4	<0.4	<2	<2
Retene	<0.4	<0.4	<0.4	<0.4	<0.4	<0.4	<2	<2
Total PAHs	**12.4**	<0.4	<0.4	**16.3**	<0.4	<0.4	<2	<2

## Data Availability

The full dataset of raw data is deposited in the Sequence Read Archive (SRA database; available at https://www.ncbi.nlm.nih.gov/sra; accession number: SUB6701449; accessed on 15 February 2021).

## Supplementary Materials

The following are available online at https://www.mdpi.com/article/10.3390/ijms22136674/s1, Table S1: Chemical analyses of total PAHs and total PCBs in sediment, Table S2: Chemical analyses of total PAHs and total PCBs in seawater, Table S3: Chemical analyses of total PAHs, total PCBs and Zn in sediment, Table S4: Chemical analyses of total PAHs, total PCBs and Zn in seawater, Supplementary Table S5: Chemical analyses of total PAHs, total PCBs and Zn in sediment, Table S6: Chemical analyses of total PAHs, total PCBs and Zn in seawater, Table S7: Chemical analyses of total PAHs, total PCBs and Zn in sediment collected at tf, Table S8: Chemical analyses of total PAHs, total PCBs and Zn in seawater collected at time zero (t1), Table S9: Adult sizes and gonadal index of adults of sea urchin *P. lividus*, Table S10: Quantity (µg/kg) of PCBs detected in thecae (including spines), gonads and gut from adult sea urchin *P. lividus*, Table S11: Number of differentially expressed (DE) genes and isoforms, Table S12: Common up-regulated genes in the Venn diagrams, Table S13: Common down-regulated genes in the Venn diagrams, Table S14: Data of expression levels in embryos at the pluteus stage, Table S15: Chemical analyses of heavy metals in sea water collected at time zero, Table S16: Chemical analyses of ammonia, nitrates, nitrites and phosphates on sea water, Table S17: Sample names, conditions and paired reads for each sample, Table S18: The number of reads obtained for the samples, Figure S1: Data of the sediment grain size analyzed with GradiStat software, Figure S2: Mortality index, Figure S3: BLASTx top-hit species distribution, Figure S4: Principal component analysis (PCA), Figure S5: Summary of the 62 genes analyzed by real-time qPCR, Figure S6: Schematic overview of *P. lividus* genes affected by artificial contaminated sediment with PAHs and PCBs under analysis, Figure S7: Schematic representation (frontal view) of 1 of the 12 experimental tanks of the mesocosms, Figure S8: Physical parameters (dissolved oxygen, pH, redox, temperature and salinity) of sea water, Figure S9: Physical parameters (dissolved oxygen, pH, redox, temperature and salinity) of sea water, Figure S10: Chemical parameters (ammonia, nitrates, nitrites and phosphates) of sea water Figure S11: Chemical parameters (ammonia, nitrates, nitrites and phosphates) of sea water.
