# Supplementary material for: Sub-Chronic Effects of Slight PAH- and PCB-Contaminated Mesocosms in Paracentrotus lividus Lmk: A Multi-Endpoint Approach and De Novo Transcriptomic
Source: Int J Mol Sci. 2021 Jun 22;22(13):6674. doi: 10.3390/ijms22136674 (PMC8268688; doi:10.3390/ijms22136674)
Supplement: Supplementary file 1 [file ijms-22-06674-s001.zip › ijms-1243960-supplementary.pdf]

Supplementary Material

Supplementary Table S1. Chemical analyses of Total PAHs and Total PCBs on sediment collected at time zero (t0, corresponding at the beginning of the experiments, before adding compounds), time one (t1, corresponding at just after adding compounds) and at the end of experiments (tf). Data are expressed as μg/L. W+SED = Water+ sediment; W+SED+PAHs = Water + sediment + PAHs; W+SED+PCBs = Water + sediment + PCBs.

	t0		t1		tf	
	**ΣPAHs (μg/L)**	**ΣPCBs (μg/L)**	**ΣPAHs (μg/L)**	**ΣPCBs (μg/L)**	**ΣPAHs (μg/L)**	**ΣPCBs (μg/L)**
**W+SED**	< 0.005	< 0.005	< 0.005	< 0.005	< 0.005	< 0.005
**W+SED+PAHs**	< 0.005	< 0.005	< 0.005	< 0.005	< 0.005	< 0.005
**W+SED+PCBs**	< 0.005	< 0.005	< 0.005	< 0.005	< 0.005	< 0.005

Supplementary Table S2. Chemical analyses of Total PAHs and Total PCBs on seawater collected at time zero (t0, corresponding at the beginning of the experiments, before adding compounds), time one (t1, corresponding at just after adding compounds) and at the end of experiments (tf). Data are expressed as μg/L. W = water; W+SED = Water+ sediment; W+SED+PAHs = Water + sediment + PAHs; W+SED+PCBs = Water + sediment + PCBs.

	**t0**		**t1**		**tf**	
	**ΣPAHs (μg/L)**	**ΣPCBs (μg/L)**	**ΣPAHs (μg/L)**	**ΣPCBs (μg/L)**	**ΣPAHs (μg/L)**	**ΣPCBs (μg/L)**
**W**	< 0.005	< 0.005	< 0.005	< 0.005	< 0.005	< 0.005
**W+SED**	< 0.005	< 0.005	< 0.005	< 0.005	< 0.005	< 0.005
**W+SED+PAHs**	< 0.005	< 0.005	< 0.005	< 0.005	< 0.005	< 0.005
**W+SED+PCBs**	< 0.005	< 0.005	< 0.005	< 0.005	< 0.005	< 0.005

Supplementary Table S3. Chemical analyses of Total PAHs, Total PCBs and Zn on sediment collected at time zero (t0), corresponding at the beginning of the experiments, before adding compounds. The samples are numbered as 1-2-3=W+SED+PAHs, 4-5-6=W+SED+PCBs, 7-8=W+SED.

(µg/L)	t0							
	1	2	3	4	5	6	7	8
**Naphthalene**	< 0.005	< 0.005	< 0.005	< 0.005	< 0.005	< 0.005	< 0.005	< 0.005
**Acenaphthylene**	< 0.005	< 0.005	< 0.005	< 0.005	< 0.005	< 0.005	< 0.005	< 0.005
**Acenaphthene**	< 0.005	< 0.005	< 0.005	< 0.005	< 0.005	< 0.005	< 0.005	< 0.005
**Fluorene**	< 0.005	< 0.005	< 0.005	< 0.005	< 0.005	< 0.005	< 0.005	< 0.005
**Anthracene**	< 0.005	< 0.005	< 0.005	< 0.005	< 0.005	< 0.005	< 0.005	< 0.005
**Phenanthrene**	< 0.005	< 0.005	< 0.005	< 0.005	< 0.005	< 0.005	< 0.005	< 0.005
**Fluoranthene**	< 0.005	< 0.005	< 0.005	< 0.005	< 0.005	< 0.005	< 0.005	< 0.005
**Pyrene**	< 0.005	< 0.005	< 0.005	< 0.005	< 0.005	< 0.005	< 0.005	< 0.005
**Benz[a]anthracene**	< 0.005	< 0.005	< 0.005	< 0.005	< 0.005	< 0.005	< 0.005	< 0.005
**Chrysene**	< 0.005	< 0.005	< 0.005	< 0.005	< 0.005	< 0.005	< 0.005	< 0.005
**Benzo(b)Fluorantene**	< 0.005	< 0.005	< 0.005	< 0.005	< 0.005	< 0.005	< 0.005	< 0.005
**Benzo[k]fluoranthene**	< 0.005	< 0.005	< 0.005	< 0.005	< 0.005	< 0.005	< 0.005	< 0.005
**Benzo[a]pyrene**	< 0.005	< 0.005	< 0.005	< 0.005	< 0.005	< 0.005	< 0.005	< 0.005
**Indeno[1,2,3-cd]pyrene**	< 0.005	< 0.005	< 0.005	< 0.005	< 0.005	< 0.005	< 0.005	< 0.005
**Dibenz[a,h]anthracene**	< 0.005	< 0.005	< 0.005	< 0.005	< 0.005	< 0.005	< 0.005	< 0.005
**Benzo[ghi]perylene**	< 0.005	< 0.005	< 0.005	< 0.005	< 0.005	< 0.005	< 0.005	< 0.005
**PCB1**	< 0.001	< 0.001	< 0.001	< 0.001	< 0.001	< 0.001	< 0.001	< 0.001
**PCB5**	< 0.001	< 0.001	< 0.001	< 0.001	< 0.001	< 0.001	< 0.001	< 0.001
**PCB18**	< 0.001	< 0.001	< 0.001	< 0.001	< 0.001	< 0.001	< 0.001	< 0.001
**PCB31**	< 0.001	< 0.001	< 0.001	< 0.001	< 0.001	< 0.001	< 0.001	< 0.001
**PCB44**	< 0.001	< 0.001	< 0.001	< 0.001	< 0.001	< 0.001	< 0.001	< 0.001
**PCB52**	< 0.001	< 0.001	< 0.001	< 0.001	< 0.001	< 0.001	< 0.001	< 0.001
**PCB66**	< 0.001	< 0.001	< 0.001	< 0.001	< 0.001	< 0.001	< 0.001	< 0.001
**PCB87**	< 0.001	< 0.001	< 0.001	< 0.001	< 0.001	< 0.001	< 0.001	< 0.001
**PCB101**	< 0.001	< 0.001	< 0.001	< 0.001	< 0.001	< 0.001	< 0.001	< 0.001
**PCB110**	< 0.001	< 0.001	< 0.001	< 0.001	< 0.001	< 0.001	< 0.001	< 0.001
**PCB138**	< 0.001	< 0.001	< 0.001	< 0.001	< 0.001	< 0.001	< 0.001	< 0.001
**PCB141**	< 0.001	< 0.001	< 0.001	< 0.001	< 0.001	< 0.001	< 0.001	< 0.001
**PCB151**	< 0.001	< 0.001	< 0.001	< 0.001	< 0.001	< 0.001	< 0.001	< 0.001
**PCB153**	< 0.001	< 0.001	< 0.001	< 0.001	< 0.001	< 0.001	< 0.001	< 0.001
**PCB170**	< 0.001	< 0.001	< 0.001	< 0.001	< 0.001	< 0.001	< 0.001	< 0.001
**PCB180**	< 0.001	< 0.001	< 0.001	< 0.001	< 0.001	< 0.001	< 0.001	< 0.001
**PCB183**	< 0.001	< 0.001	< 0.001	< 0.001	< 0.001	< 0.001	< 0.001	< 0.001
**PCB187**	< 0.001	< 0.001	< 0.001	< 0.001	< 0.001	< 0.001	< 0.001	< 0.001
**PCB206**	< 0.001	< 0.001	< 0.001	< 0.001	< 0.001	< 0.001	< 0.001	< 0.001

Supplementary Table S4. Chemical analyses of Total PAHs, Total PCBs and Zn on seawater collected at time zero (t0), corresponding at the beginning of the experiments, before adding compounds. The samples are numbered as 1-2-3=W+SED+PAHs, 4-5-6=W+SED+PCBs, 7-8=W+SED, 9-10=W.

	**t0**									
**(µg/L)**	**1**	**2**	**3**	**4**	**5**	**6**	**7**	**8**	**9**	**10**
**Naphthalene**	< 0.05	< 0.05	< 0.05	< 0.05	< 0.05	< 0.05	< 0.05	< 0.05	< 0.05	< 0.05
**Acenaphthylene**	< 0.05	< 0.05	< 0.05	< 0.05	< 0.05	< 0.05	< 0.05	< 0.05	< 0.05	< 0.05
**Acenaphthene**	< 0.05	< 0.05	< 0.05	< 0.05	< 0.05	< 0.05	< 0.05	< 0.05	< 0.05	< 0.05
**Fluorene**	< 0.05	< 0.05	< 0.05	< 0.05	< 0.05	< 0.05	< 0.05	< 0.05	< 0.05	< 0.05
**Anthracene**	< 0.05	< 0.05	< 0.05	< 0.05	< 0.05	< 0.05	< 0.05	< 0.05	< 0.05	< 0.05
**Phenanthrene**	< 0.05	< 0.05	< 0.05	< 0.05	< 0.05	< 0.05	< 0.05	< 0.05	< 0.05	< 0.05
**Fluoranthene**	< 0.05	< 0.05	< 0.05	< 0.05	< 0.05	< 0.05	< 0.05	< 0.05	< 0.05	< 0.05
**Pyrene**	< 0.05	< 0.05	< 0.05	< 0.05	< 0.05	< 0.05	< 0.05	< 0.05	< 0.05	< 0.05
**Benz[a]anthracene**	< 0.05	< 0.05	< 0.05	< 0.05	< 0.05	< 0.05	< 0.05	< 0.05	< 0.05	< 0.05
**Chrysene**	< 0.05	< 0.05	< 0.05	< 0.05	< 0.05	< 0.05	< 0.05	< 0.05	< 0.05	< 0.05
**Benzo(b)Fluorantene**	< 0.05	< 0.05	< 0.05	< 0.05	< 0.05	< 0.05	< 0.05	< 0.05	< 0.05	< 0.05
**Benzo[k]fluoranthene**	< 0.05	< 0.05	< 0.05	< 0.05	< 0.05	< 0.05	< 0.05	< 0.05	< 0.05	< 0.05
**Benzo[a]pyrene**	< 0.05	< 0.05	< 0.05	< 0.05	< 0.05	< 0.05	< 0.05	< 0.05	< 0.05	< 0.05
**Indeno[1,2,3-cd]pyrene**	< 0.05	< 0.05	< 0.05	< 0.05	< 0.05	< 0.05	< 0.05	< 0.05	< 0.05	< 0.05
**Dibenz[a,h]anthracene**	< 0.05	< 0.05	< 0.05	< 0.05	< 0.05	< 0.05	< 0.05	< 0.05	< 0.05	< 0.05
**Benzo[ghi]perylene**	< 0.05	< 0.05	< 0.05	< 0.05	< 0.05	< 0.05	< 0.05	< 0.05	< 0.05	< 0.05
**PCB1**	< 0.05	< 0.05	< 0.05	< 0.05	< 0.05	< 0.05	< 0.05	< 0.05	< 0.05	< 0.05
**PCB5**	< 0.05	< 0.05	< 0.05	< 0.05	< 0.05	< 0.05	< 0.05	< 0.05	< 0.05	< 0.05
**PCB18**	< 0.05	< 0.05	< 0.05	< 0.05	< 0.05	< 0.05	< 0.05	< 0.05	< 0.05	< 0.05
**PCB31**	< 0.05	< 0.05	< 0.05	< 0.05	< 0.05	< 0.05	< 0.05	< 0.05	< 0.05	< 0.05
**PCB44**	< 0.05	< 0.05	< 0.05	< 0.05	< 0.05	< 0.05	< 0.05	< 0.05	< 0.05	< 0.05
**PCB52**	< 0.05	< 0.05	< 0.05	< 0.05	< 0.05	< 0.05	< 0.05	< 0.05	< 0.05	< 0.05
**PCB66**	< 0.05	< 0.05	< 0.05	< 0.05	< 0.05	< 0.05	< 0.05	< 0.05	< 0.05	< 0.05
**PCB87**	< 0.05	< 0.05	< 0.05	< 0.05	< 0.05	< 0.05	< 0.05	< 0.05	< 0.05	< 0.05
**PCB101**	< 0.05	< 0.05	< 0.05	< 0.05	< 0.05	< 0.05	< 0.05	< 0.05	< 0.05	< 0.05
**PCB110**	< 0.05	< 0.05	< 0.05	< 0.05	< 0.05	< 0.05	< 0.05	< 0.05	< 0.05	< 0.05
**PCB138**	< 0.05	< 0.05	< 0.05	< 0.05	< 0.05	< 0.05	< 0.05	< 0.05	< 0.05	< 0.05
**PCB141**	< 0.05	< 0.05	< 0.05	< 0.05	< 0.05	< 0.05	< 0.05	< 0.05	< 0.05	< 0.05
**PCB151**	< 0.05	< 0.05	< 0.05	< 0.05	< 0.05	< 0.05	< 0.05	< 0.05	< 0.05	< 0.05
**PCB153**	< 0.05	< 0.05	< 0.05	< 0.05	< 0.05	< 0.05	< 0.05	< 0.05	< 0.05	< 0.05
**PCB170**	< 0.05	< 0.05	< 0.05	< 0.05	< 0.05	< 0.05	< 0.05	< 0.05	< 0.05	< 0.05
**PCB180**	< 0.05	< 0.05	< 0.05	< 0.05	< 0.05	< 0.05	< 0.05	< 0.05	< 0.05	< 0.05
**PCB183**	< 0.05	< 0.05	< 0.05	< 0.05	< 0.05	< 0.05	< 0.05	< 0.05	< 0.05	< 0.05
**PCB187**	< 0.05	< 0.05	< 0.05	< 0.05	< 0.05	< 0.05	< 0.05	< 0.05	< 0.05	< 0.05
**PCB206**	< 0.05	< 0.05	< 0.05	< 0.05	< 0.05	< 0.05	< 0.05	< 0.05	< 0.05	< 0.05

Supplementary Table S5. Chemical analyses of Total PAHs, Total PCBs and Zn on sediment collected at time one (t1), corresponding just after adding compounds. The samples are numbered as 1-2-3=W+SED+PAHs, 4-5-6=W+SED+PCBs, 7-8=W+SED.

(µg/L)	t1							
	**1**	**2**	**3**	**4**	**5**	**6**	**7**	**8**
**Naphthalene**	< 0.005	< 0.005	< 0.005	< 0.005	< 0.005	< 0.005	< 0.005	< 0.005
**Acenaphthylene**	< 0.005	< 0.005	< 0.005	< 0.005	< 0.005	< 0.005	< 0.005	< 0.005
**Acenaphthene**	< 0.005	< 0.005	< 0.005	< 0.005	< 0.005	< 0.005	< 0.005	< 0.005
**Fluorene**	< 0.005	< 0.005	< 0.005	< 0.005	< 0.005	< 0.005	< 0.005	< 0.005
**Anthracene**	< 0.005	< 0.005	< 0.005	< 0.005	< 0.005	< 0.005	< 0.005	< 0.005
**Phenanthrene**	< 0.005	< 0.005	< 0.005	< 0.005	< 0.005	< 0.005	< 0.005	< 0.005
**Fluoranthene**	< 0.005	< 0.005	< 0.005	< 0.005	< 0.005	< 0.005	< 0.005	< 0.005
**Pyrene**	< 0.005	< 0.005	< 0.005	< 0.005	< 0.005	< 0.005	< 0.005	< 0.005
**Benz[a]anthracene**	< 0.005	< 0.005	< 0.005	< 0.005	< 0.005	< 0.005	< 0.005	< 0.005
**Chrysene**	< 0.005	< 0.005	< 0.005	< 0.005	< 0.005	< 0.005	< 0.005	< 0.005
**Benzo(b)Fluorantene**	< 0.005	< 0.005	< 0.005	< 0.005	< 0.005	< 0.005	< 0.005	< 0.005
**Benzo[k]fluoranthene**	< 0.005	< 0.005	< 0.005	< 0.005	< 0.005	< 0.005	< 0.005	< 0.005
**Benzo[a]pyrene**	< 0.005	< 0.005	< 0.005	< 0.005	< 0.005	< 0.005	< 0.005	< 0.005
**Indeno[1,2,3-cd]pyrene**	< 0.005	< 0.005	< 0.005	< 0.005	< 0.005	< 0.005	< 0.005	< 0.005
**Dibenz[a,h]anthracene**	< 0.005	< 0.005	< 0.005	< 0.005	< 0.005	< 0.005	< 0.005	< 0.005
**Benzo[ghi]perylene**	< 0.005	< 0.005	< 0.005	< 0.005	< 0.005	< 0.005	< 0.005	< 0.005
**PCB1**	< 0.001	< 0.001	< 0.001	< 0.001	< 0.001	< 0.001	< 0.001	< 0.001
**PCB5**	< 0.001	< 0.001	< 0.001	< 0.001	< 0.001	< 0.001	< 0.001	< 0.001
**PCB18**	< 0.001	< 0.001	< 0.001	< 0.001	< 0.001	< 0.001	< 0.001	< 0.001
**PCB31**	< 0.001	< 0.001	< 0.001	< 0.001	< 0.001	< 0.001	< 0.001	< 0.001
**PCB44**	< 0.001	< 0.001	< 0.001	< 0.001	< 0.001	< 0.001	< 0.001	< 0.001
**PCB52**	< 0.001	< 0.001	< 0.001	< 0.001	< 0.001	< 0.001	< 0.001	< 0.001
**PCB66**	< 0.001	< 0.001	< 0.001	< 0.001	< 0.001	< 0.001	< 0.001	< 0.001
**PCB87**	< 0.001	< 0.001	< 0.001	< 0.001	< 0.001	< 0.001	< 0.001	< 0.001
**PCB101**	< 0.001	< 0.001	< 0.001	< 0.001	< 0.001	< 0.001	< 0.001	< 0.001
**PCB110**	< 0.001	< 0.001	< 0.001	< 0.001	< 0.001	< 0.001	< 0.001	< 0.001
**PCB138**	< 0.001	< 0.001	< 0.001	< 0.001	< 0.001	< 0.001	< 0.001	< 0.001
**PCB141**	< 0.001	< 0.001	< 0.001	< 0.001	< 0.001	< 0.001	< 0.001	< 0.001
**PCB151**	< 0.001	< 0.001	< 0.001	< 0.001	< 0.001	< 0.001	< 0.001	< 0.001
**PCB153**	< 0.001	< 0.001	< 0.001	< 0.001	< 0.001	< 0.001	< 0.001	< 0.001
**PCB170**	< 0.001	< 0.001	< 0.001	< 0.001	< 0.001	< 0.001	< 0.001	< 0.001
**PCB180**	< 0.001	< 0.001	< 0.001	< 0.001	< 0.001	< 0.001	< 0.001	< 0.001
**PCB183**	< 0.001	< 0.001	< 0.001	< 0.001	< 0.001	< 0.001	< 0.001	< 0.001
**PCB187**	< 0.001	< 0.001	< 0.001	< 0.001	< 0.001	< 0.001	< 0.001	< 0.001
**PCB206**	< 0.001	< 0.001	< 0.001	< 0.001	< 0.001	< 0.001	< 0.001	< 0.001

Supplementary Table S6. Chemical analyses of Total PAHs, Total PCBs and Zn on seawater collected at time zero (t1), corresponding just after adding compounds. The samples are numbered as 1-2-3=W+SED+PAHs, 4-5-6=W+SED+PCBs, 7-8=W+SED, 9-10=W.

	t1									
**(μg/L)**	**1**	**2**	**3**	**4**	**5**	**6**	**7**	**8**	**9**	**10**
**Naphthalene**	< 0.05	< 0.05	< 0.05	< 0.05	< 0.05	< 0.05	< 0.05	< 0.05	< 0.05	< 0.05
**Acenaphthylene**	< 0.05	< 0.05	< 0.05	< 0.05	< 0.05	< 0.05	< 0.05	< 0.05	< 0.05	< 0.05
**Acenaphthene**	< 0.05	< 0.05	< 0.05	< 0.05	< 0.05	< 0.05	< 0.05	< 0.05	< 0.05	< 0.05
**Fluorene**	< 0.05	< 0.05	< 0.05	< 0.05	< 0.05	< 0.05	< 0.05	< 0.05	< 0.05	< 0.05
**Anthracene**	< 0.05	< 0.05	< 0.05	< 0.05	< 0.05	< 0.05	< 0.05	< 0.05	< 0.05	< 0.05
**Phenanthrene**	< 0.05	< 0.05	< 0.05	< 0.05	< 0.05	< 0.05	< 0.05	< 0.05	< 0.05	< 0.05
**Fluoranthene**	< 0.05	< 0.05	< 0.05	< 0.05	< 0.05	< 0.05	< 0.05	< 0.05	< 0.05	< 0.05
**Pyrene**	< 0.05	< 0.05	< 0.05	< 0.05	< 0.05	< 0.05	< 0.05	< 0.05	< 0.05	< 0.05
**Benz[a]anthracene**	< 0.05	< 0.05	< 0.05	< 0.05	< 0.05	< 0.05	< 0.05	< 0.05	< 0.05	< 0.05
**Chrysene**	< 0.05	< 0.05	< 0.05	< 0.05	< 0.05	< 0.05	< 0.05	< 0.05	< 0.05	< 0.05
**Benzo(b)Fluorantene**	< 0.05	< 0.05	< 0.05	< 0.05	< 0.05	< 0.05	< 0.05	< 0.05	< 0.05	< 0.05
**Benzo[k]fluoranthene**	< 0.05	< 0.05	< 0.05	< 0.05	< 0.05	< 0.05	< 0.05	< 0.05	< 0.05	< 0.05
**Benzo[a]pyrene**	< 0.05	< 0.05	< 0.05	< 0.05	< 0.05	< 0.05	< 0.05	< 0.05	< 0.05	< 0.05
**Indeno[1,2,3-cd]pyrene**	< 0.05	< 0.05	< 0.05	< 0.05	< 0.05	< 0.05	< 0.05	< 0.05	< 0.05	< 0.05
**Dibenz[a,h]anthracene**	< 0.05	< 0.05	< 0.05	< 0.05	< 0.05	< 0.05	< 0.05	< 0.05	< 0.05	< 0.05
**Benzo[ghi]perylene**	< 0.05	< 0.05	< 0.05	< 0.05	< 0.05	< 0.05	< 0.05	< 0.05	< 0.05	< 0.05
**PCB1**	< 0.05	< 0.05	< 0.05	< 0.05	< 0.05	< 0.05	< 0.05	< 0.05	< 0.05	< 0.05
**PCB5**	< 0.05	< 0.05	< 0.05	< 0.05	< 0.05	< 0.05	< 0.05	< 0.05	< 0.05	< 0.05
**PCB18**	< 0.05	< 0.05	< 0.05	< 0.05	< 0.05	< 0.05	< 0.05	< 0.05	< 0.05	< 0.05

**PCB31**	< 0.05	< 0.05	< 0.05	< 0.05	< 0.05	< 0.05	< 0.05	< 0.05	< 0.05	< 0.05
**PCB44**	< 0.05	< 0.05	< 0.05	< 0.05	< 0.05	< 0.05	< 0.05	< 0.05	< 0.05	< 0.05
**PCB52**	< 0.05	< 0.05	< 0.05	< 0.05	< 0.05	< 0.05	< 0.05	< 0.05	< 0.05	< 0.05
**PCB66**	< 0.05	< 0.05	< 0.05	< 0.05	< 0.05	< 0.05	< 0.05	< 0.05	< 0.05	< 0.05
**PCB87**	< 0.05	< 0.05	< 0.05	< 0.05	< 0.05	< 0.05	< 0.05	< 0.05	< 0.05	< 0.05
**PCB101**	< 0.05	< 0.05	< 0.05	< 0.05	< 0.05	< 0.05	< 0.05	< 0.05	< 0.05	< 0.05
**PCB110**	< 0.05	< 0.05	< 0.05	< 0.05	< 0.05	< 0.05	< 0.05	< 0.05	< 0.05	< 0.05
**PCB138**	< 0.05	< 0.05	< 0.05	< 0.05	< 0.05	< 0.05	< 0.05	< 0.05	< 0.05	< 0.05
**PCB141**	< 0.05	< 0.05	< 0.05	< 0.05	< 0.05	< 0.05	< 0.05	< 0.05	< 0.05	< 0.05
**PCB151**	< 0.05	< 0.05	< 0.05	< 0.05	< 0.05	< 0.05	< 0.05	< 0.05	< 0.05	< 0.05
**PCB153**	< 0.05	< 0.05	< 0.05	< 0.05	< 0.05	< 0.05	< 0.05	< 0.05	< 0.05	< 0.05
**PCB170**	< 0.05	< 0.05	< 0.05	< 0.05	< 0.05	< 0.05	< 0.05	< 0.05	< 0.05	< 0.05
**PCB180**	< 0.05	< 0.05	< 0.05	< 0.05	< 0.05	< 0.05	< 0.05	< 0.05	< 0.05	< 0.05
**PCB183**	< 0.05	< 0.05	< 0.05	< 0.05	< 0.05	< 0.05	< 0.05	< 0.05	< 0.05	< 0.05
**PCB187**	< 0.05	< 0.05	< 0.05	< 0.05	< 0.05	< 0.05	< 0.05	< 0.05	< 0.05	< 0.05
**PCB206**	< 0.05	< 0.05	< 0.05	< 0.05	< 0.05	< 0.05	< 0.05	< 0.05	< 0.05	< 0.05

Supplementary Table S7. Chemical analyses of Total PAHs, Total PCBs and Zn on sediment collected at tf, corresponding at the end of the experiments. The samples are numbered as 1-2-3=W+SED+PAHs, 4-5-6=W+SED+PCBs, 7-8=W+SED.

(µg/L)	tf							
	**1**	**2**	**3**	**4**	**5**	**6**	**7**	**8**
**Naphthalene**	< 0.005	< 0.005	< 0.005	< 0.005	< 0.005	< 0.005	< 0.005	< 0.005
**Acenaphthylene**	< 0.005	< 0.005	< 0.005	< 0.005	< 0.005	< 0.005	< 0.005	< 0.005
**Acenaphthene**	< 0.005	< 0.005	< 0.005	< 0.005	< 0.005	< 0.005	< 0.005	< 0.005
**Fluorene**	< 0.005	< 0.005	< 0.005	< 0.005	< 0.005	< 0.005	< 0.005	< 0.005
**Anthracene**	< 0.005	< 0.005	< 0.005	< 0.005	< 0.005	< 0.005	< 0.005	< 0.005
**Phenanthrene**	< 0.005	< 0.005	< 0.005	< 0.005	< 0.005	< 0.005	< 0.005	< 0.005
**Fluoranthene**	< 0.005	< 0.005	< 0.005	< 0.005	< 0.005	< 0.005	< 0.005	< 0.005
**Pyrene**	< 0.005	< 0.005	< 0.005	< 0.005	< 0.005	< 0.005	< 0.005	< 0.005
**Benz[a]anthracene**	< 0.005	< 0.005	< 0.005	< 0.005	< 0.005	< 0.005	< 0.005	< 0.005
**Chrysene**	< 0.005	< 0.005	< 0.005	< 0.005	< 0.005	< 0.005	< 0.005	< 0.005
**Benzo(b)Fluorantene**	< 0.005	< 0.005	< 0.005	< 0.005	< 0.005	< 0.005	< 0.005	< 0.005
**Benzo[k]fluoranthene**	< 0.005	< 0.005	< 0.005	< 0.005	< 0.005	< 0.005	< 0.005	< 0.005
**Benzo[a]pyrene**	< 0.005	< 0.005	< 0.005	< 0.005	< 0.005	< 0.005	< 0.005	< 0.005
**Indeno[1,2,3-cd]pyrene**	< 0.005	< 0.005	< 0.005	< 0.005	< 0.005	< 0.005	< 0.005	< 0.005
**Dibenz[a,h]anthracene**	< 0.005	< 0.005	< 0.005	< 0.005	< 0.005	< 0.005	< 0.005	< 0.005
**Benzo[ghi]perylene**	< 0.005	< 0.005	< 0.005	< 0.005	< 0.005	< 0.005	< 0.005	< 0.005
**PCB1**	< 0.001	< 0.001	< 0.001	< 0.001	< 0.001	< 0.001	< 0.001	< 0.001
**PCB5**	< 0.001	< 0.001	< 0.001	< 0.001	< 0.001	< 0.001	< 0.001	< 0.001
**PCB18**	< 0.001	< 0.001	< 0.001	< 0.001	< 0.001	< 0.001	< 0.001	< 0.001
**PCB31**	< 0.001	< 0.001	< 0.001	< 0.001	< 0.001	< 0.001	< 0.001	< 0.001
**PCB44**	< 0.001	< 0.001	< 0.001	< 0.001	< 0.001	< 0.001	< 0.001	< 0.001
**PCB52**	< 0.001	< 0.001	< 0.001	< 0.001	< 0.001	< 0.001	< 0.001	< 0.001
**PCB66**	< 0.001	< 0.001	< 0.001	< 0.001	< 0.001	< 0.001	< 0.001	< 0.001
**PCB87**	< 0.001	< 0.001	< 0.001	< 0.001	< 0.001	< 0.001	< 0.001	< 0.001
**PCB101**	< 0.001	< 0.001	< 0.001	< 0.001	< 0.001	< 0.001	< 0.001	< 0.001
**PCB110**	< 0.001	< 0.001	< 0.001	< 0.001	< 0.001	< 0.001	< 0.001	< 0.001
**PCB138**	< 0.001	< 0.001	< 0.001	< 0.001	< 0.001	< 0.001	< 0.001	< 0.001
**PCB141**	< 0.001	< 0.001	< 0.001	< 0.001	< 0.001	< 0.001	< 0.001	< 0.001
**PCB151**	< 0.001	< 0.001	< 0.001	< 0.001	< 0.001	< 0.001	< 0.001	< 0.001
**PCB153**	< 0.001	< 0.001	< 0.001	< 0.001	< 0.001	< 0.001	< 0.001	< 0.001
**PCB170**	< 0.001	< 0.001	< 0.001	< 0.001	< 0.001	< 0.001	< 0.001	< 0.001
**PCB180**	< 0.001	< 0.001	< 0.001	< 0.001	< 0.001	< 0.001	< 0.001	< 0.001
**PCB183**	< 0.001	< 0.001	< 0.001	< 0.001	< 0.001	< 0.001	< 0.001	< 0.001
**PCB187**	< 0.001	< 0.001	< 0.001	< 0.001	< 0.001	< 0.001	< 0.001	< 0.001
**PCB206**	< 0.001	< 0.001	< 0.001	< 0.001	< 0.001	< 0.001	< 0.001	< 0.001

Supplementary Table S8. Chemical analyses of Total PAHs, Total PCBs and Zn on seawater collected at time zero (t^1), corresponding at the end of the experiments. The samples are numbered as 1-2-3=W+SED+PAHs, 4-5-6=W+SED+PCBs, 7-8=W+SED, 9-10=W.

(µg/L)	tf									
	1	2	3	4	5	6	7	8	9	10
**Naphthalene**	< 0.05	< 0.05	< 0.05	< 0.05	< 0.05	< 0.05	< 0.05	< 0.05	< 0.05	< 0.05
**Acenaphthylene**	< 0.05	< 0.05	< 0.05	< 0.05	< 0.05	< 0.05	< 0.05	< 0.05	< 0.05	< 0.05
**Acenaphthene**	< 0.05	< 0.05	< 0.05	< 0.05	< 0.05	< 0.05	< 0.05	< 0.05	< 0.05	< 0.05
**Fluorene**	< 0.05	< 0.05	< 0.05	< 0.05	< 0.05	< 0.05	< 0.05	< 0.05	< 0.05	< 0.05
**Anthracene**	< 0.05	< 0.05	< 0.05	< 0.05	< 0.05	< 0.05	< 0.05	< 0.05	< 0.05	< 0.05
**Phenanthrene**	< 0.05	< 0.05	< 0.05	< 0.05	< 0.05	< 0.05	< 0.05	< 0.05	< 0.05	< 0.05
**Fluoranthene**	< 0.05	< 0.05	< 0.05	< 0.05	< 0.05	< 0.05	< 0.05	< 0.05	< 0.05	< 0.05
**Pyrene**	< 0.05	< 0.05	< 0.05	< 0.05	< 0.05	< 0.05	< 0.05	< 0.05	< 0.05	< 0.05
**Benz[a]anthracene**	< 0.05	< 0.05	< 0.05	< 0.05	< 0.05	< 0.05	< 0.05	< 0.05	< 0.05	< 0.05
**Chrysene**	< 0.05	< 0.05	< 0.05	< 0.05	< 0.05	< 0.05	< 0.05	< 0.05	< 0.05	< 0.05
**Benzo(b)Fluorantene**	< 0.05	< 0.05	< 0.05	< 0.05	< 0.05	< 0.05	< 0.05	< 0.05	< 0.05	< 0.05
**Benzo[k]fluoranthene**	< 0.05	< 0.05	< 0.05	< 0.05	< 0.05	< 0.05	< 0.05	< 0.05	< 0.05	< 0.05
**Benzo[a]pyrene**	< 0.05	< 0.05	< 0.05	< 0.05	< 0.05	< 0.05	< 0.05	< 0.05	< 0.05	< 0.05
**Indeno[1,2,3-cd]pyrene**	< 0.05	< 0.05	< 0.05	< 0.05	< 0.05	< 0.05	< 0.05	< 0.05	< 0.05	< 0.05
**Dibenz[a,h]anthracene**	< 0.05	< 0.05	< 0.05	< 0.05	< 0.05	< 0.05	< 0.05	< 0.05	< 0.05	< 0.05
**Benzo[ghi]perylene**	< 0.05	< 0.05	< 0.05	< 0.05	< 0.05	< 0.05	< 0.05	< 0.05	< 0.05	< 0.05
**PCB1**	< 0.05	< 0.05	< 0.05	< 0.05	< 0.05	< 0.05	< 0.05	< 0.05	< 0.05	< 0.05
**PCB5**	< 0.05	< 0.05	< 0.05	< 0.05	< 0.05	< 0.05	< 0.05	< 0.05	< 0.05	< 0.05
**PCB18**	< 0.05	< 0.05	< 0.05	< 0.05	< 0.05	< 0.05	< 0.05	< 0.05	< 0.05	< 0.05
**PCB31**	< 0.05	< 0.05	< 0.05	< 0.05	< 0.05	< 0.05	< 0.05	< 0.05	< 0.05	< 0.05
**PCB44**	< 0.05	< 0.05	< 0.05	< 0.05	< 0.05	< 0.05	< 0.05	< 0.05	< 0.05	< 0.05
**PCB52**	< 0.05	< 0.05	< 0.05	< 0.05	< 0.05	< 0.05	< 0.05	< 0.05	< 0.05	< 0.05
**PCB66**	< 0.05	< 0.05	< 0.05	< 0.05	< 0.05	< 0.05	< 0.05	< 0.05	< 0.05	< 0.05
**PCB87**	< 0.05	< 0.05	< 0.05	< 0.05	< 0.05	< 0.05	< 0.05	< 0.05	< 0.05	< 0.05
**PCB101**	< 0.05	< 0.05	< 0.05	< 0.05	< 0.05	< 0.05	< 0.05	< 0.05	< 0.05	< 0.05
**PCB110**	< 0.05	< 0.05	< 0.05	< 0.05	< 0.05	< 0.05	< 0.05	< 0.05	< 0.05	< 0.05
**PCB138**	< 0.05	< 0.05	< 0.05	< 0.05	< 0.05	< 0.05	< 0.05	< 0.05	< 0.05	< 0.05
**PCB141**	< 0.05	< 0.05	< 0.05	< 0.05	< 0.05	< 0.05	< 0.05	< 0.05	< 0.05	< 0.05
**PCB151**	< 0.05	< 0.05	< 0.05	< 0.05	< 0.05	< 0.05	< 0.05	< 0.05	< 0.05	< 0.05
**PCB153**	< 0.05	< 0.05	< 0.05	< 0.05	< 0.05	< 0.05	< 0.05	< 0.05	< 0.05	< 0.05
**PCB170**	< 0.05	< 0.05	< 0.05	< 0.05	< 0.05	< 0.05	< 0.05	< 0.05	< 0.05	< 0.05
**PCB180**	< 0.05	< 0.05	< 0.05	< 0.05	< 0.05	< 0.05	< 0.05	< 0.05	< 0.05	< 0.05
**PCB183**	< 0.05	< 0.05	< 0.05	< 0.05	< 0.05	< 0.05	< 0.05	< 0.05	< 0.05	< 0.05
**PCB187**	< 0.05	< 0.05	< 0.05	< 0.05	< 0.05	< 0.05	< 0.05	< 0.05	< 0.05	< 0.05
**PCB206**	< 0.05	< 0.05	< 0.05	< 0.05	< 0.05	< 0.05	< 0.05	< 0.05	< 0.05	< 0.05

Supplementary Table S9. Adult sizes (in millimetres) and gonadal index (GI ± SD, n = 5/group) of adults of sea urchin *P. lividus* collected in the field at the beginning (t0) and after two months in the three experimental conditions: W, W+SED, W+SED+PAHs and W+SED+PCBs (p value > 0.05). W = water; W+SED = Water+ sediment; W+SED+PAHs = Water + sediment + PAHs; W+SED+PCBs = Water + sediment + PCBs.

		**W**	**W+SED**	**W+SED+ PAHs**	**W+SED+PCBs**
**Adult size,**	50.5 ± 4.36				
**t_0**		44.5 ± 1.1	51.9 ± 5.6	58.8 ± 5.3	56.6 ± 3.7
**2 months**		37.2 ± 9.6	52.2 ± 5.1	57.6 ± 5.9	56.1 ± 3.9
**GI**					
**t_0**	3.68 ± 0.01				
**2 months**		0.88 ± 0.01	3.78 ± 0.02	2.32 ± 0.02	2.22 ± 0.004

Supplementary Table S10. Quantity (μg/Kg) PCBs detected in theca including spines, gonads and gut from adult sea urchin *P. lividus* in two experimental conditions after two months: W/W+SED, W+SED+PCBs.

	Theca + spines		Gonads		Gut	
**PCBs**	**W+SED+PCBs**	**W+SED/W**	**W+SED+PCBs**	**W+SED/W**	**W+SED+PCBs**	**W+SED/W**
**PCB1**	< 0.5	< 0.5	< 2	< 2	< 2	< 2
**PCB5**	< 0.5	< 0.5	< 2	< 2	< 2	< 2
**PCB18**	< 0.5	< 0.5	< 2	< 2	< 2	< 2
**PCB31**	< 0.5	< 0.5	< 2	< 2	< 2	< 2
**PCB44**	< 0.5	< 0.5	< 2	< 2	< 2	< 2
**PCB52**	< 0.5	< 0.5	< 2	< 2	< 2	< 2
**PCB66**	< 0.5	< 0.5	< 2	< 2	< 2	< 2
**PCB87**	< 0.5	< 0.5	< 2	< 2	< 2	< 2
**PCB101**	< 0.5	< 0.5	< 2	< 2	< 2	< 2
**PCB110**	< 0.5	< 0.5	< 2	< 2	< 2	< 2
**PCB138**	< 0.5	< 0.5	< 2	< 2	< 2	< 2
**PCB141**	< 0.5	< 0.5	< 2	< 2	< 2	< 2
**PCB151**	< 0.5	< 0.5	< 2	< 2	< 2	< 2
**PCB153**	< 0.5	< 0.5	< 2	< 2	< 2	< 2
**PCB170**	< 0.5	< 0.5	< 2	< 2	< 2	< 2
**PCB180**	< 0.5	< 0.5	< 2	< 2	< 2	< 2
**PCB183**	< 0.5	< 0.5	< 2	< 2	< 2	< 2
**PCB187**	< 0.5	< 0.5	< 2	< 2	< 2	< 2
**PCB206**	< 0.5	< 0.5	< 2	< 2	< 2	< 2
**Total PCBs**	< 0.5	< 0.5	< 2	< 2	< 2	< 2

Supplementary Table S11. Number of differentially expressed (DE) genes and isoforms, identified with a False Discovery Rate (FDR) ≤ 0.05, and with a Fold Change (FC) ≥ 1.5 (upregulated genes/isoforms) and ≤ −1.5 (down-regulated genes/isoforms).Control: plutei from adults sea urchin *P. lividus* reared for two months in tanks with sediment without contaminants; Treated1: plutei deriving from adults exposed for two months to sediment contaminated with PAHs; Treated2: plutei deriving from adults exposed for two months to sediment contaminated with PCBs.

	**FDR ≤ 0.05**	**FC ≥ 1.5 FDR ≤ 0.05**	**FC ≤ -1.5 FDR ≤ 0.05**
**Genes**			
**Treated1 vs Control**	1898	933	965
**Treated2_ vs Control**	2396	1079	1317
**Treated2 vs Treared1**	1356	755	601
**Isoforms**			
**Treated1 vs Control**	5591	2200	3388
**Treated2_ vs Control**	7703	3715	3985
**Treated2 vs Treared1**	4762	2911	1850

Supplementary Table S12. Common up-regulated genes in the Venn diagrams (for more details see also **Figure 4** and legend to this figure) among the three experimental groups: "Treated_1 (plutei deriving from adults exposed for two months to sediment contaminated with PAHs) versus Control" (plutei from adults sea urchin *P. lividus* reared for two months in tanks with sediment without contaminants, "Treated_2 (plutei deriving from adults exposed for two months to sediment contaminated with PCBSs) versus Control" and "Treated_1 versus Treated_2".

**Up-regulated genes**
**"Treated_1 vs Control" and "Treated_2 vs Control"**
DNA replication licensing factor mcm7-A isoform X1
unnamed protein product
diamine acetyltransferase 2
arylsulfatase
PREDICTED: uncharacterized protein LOC578221
high-affinity choline transporter 1
PREDICTED: uncharacterized protein LOC100889675
PREDICTED: fibropellin-3
PREDICTED: uncharacterized protein LOC100891625
nucleolin isoform X3
PREDICTED: titin
spectrin alpha chain, non-erythrocytic 1 isoform X6
185/333
tethering factor for nuclear proteasome STS1 isoform X2
tryptophan 5-hydroxylase 1 isoform X1
high-affinity choline transporter 1-like
DYI2_HELCRRecName: Full=Dynein intermediate chain 2, ciliary
hypothetical protein B7P43_G03953
putative nuclease HARBI1
dolichyl-diphosphooligosaccharide--protein glycosyltransferase subunit 1
nodal
putative TBC1 domain family member 2A
PREDICTED: uncharacterized protein LOC105436430
threonine synthase-like 1
E3 ubiquitin-protein ligase RNF8 isoform X1
PREDICTED: trypsin-1-like
uncharacterized protein LOC111859709
PREDICTED: uncharacterized protein LOC575796
MFS-type transporter SLC18B1
antivin/lefty
deleted in malignant brain tumors 1 protein
PREDICTED: uncharacterized protein LOC105441695
PREDICTED: uncharacterized protein LOC100888967
hypothetical protein pdam_00008083
cell wall-associated hydrolase

alpha-5 collagen
retinol dehydrogenase 8
MYCBP-associated protein isoform X4
heparan sulfate 2-O-sulfotransferase 1
PREDICTED: uncharacterized protein LOC100889612
PREDICTED: uncharacterized protein LOC754545
PREDICTED: uncharacterized protein LOC756027
uncharacterized protein LOC113054557
condensin complex subunit 3 isoform X1
PREDICTED: uncharacterized protein LOC105444166
transmembrane 9 superfamily member 4
PREDICTED: uncharacterized protein LOC105439689
PREDICTED: uncharacterized protein LOC764367
DNMT1_PARLIRecName: (cytosine-5)-methyltransferase PliMCI
NFU1 iron-sulfur cluster scaffold homolog, mitochondrial
hypothetical protein BSL78_07137, partial
PREDICTED: uncharacterized protein LOC108671849
forkhead transcription factor Q2
ubiquitin carboxyl-terminal hydrolase CYLD
uncharacterized protein K02A2.6-like
hypothetical protein X975_19031, partial
PREDICTED: uncharacterized protein LOC105447528
putative RNA-directed DNA polymerase from transposon BS
PREDICTED: uncharacterized protein LOC105439906
PREDICTED: uncharacterized protein LOC105442605
PREDICTED: uncharacterized protein LOC100890383
reverse transcriptase family protein
dnaJ homolog subfamily B member 13
laminin subunit alpha
meiosis-specific protein MEI4-like
uncharacterized protein LOC114575677
PREDICTED: uncharacterized protein LOC100892571
hypothetical protein BBROOKSOX_704
PREDICTED: uncharacterized protein LOC105444429
PREDICTED: uncharacterized protein LOC105326080
long-chain-fatty-acid--CoA ligase 4
coiled-coil domain-containing protein 40
PREDICTED: uncharacterized protein LOC590579
PREDICTED: uncharacterized protein LOC105335220

**"Treated_1 vs Control", "Treated_2 vs Control" and "Treated_1 vs Treated_2"**
putative RNA-directed DNA polymerase from transposon X-element
PREDICTED: uncharacterized protein LOC100890353

hypothetical protein
PREDICTED: uncharacterized protein LOC105439418
RNA-directed DNA polymerase from mobile element jockey-like
PREDICTED: uncharacterized protein LOC105444767
protein FAM166B
uncharacterized protein LOC110990766
PREDICTED: uncharacterized protein K02A2.6-like
PREDICTED: uncharacterized protein LOC105440140
PREDICTED: uncharacterized protein LOC587493
PREDICTED: uncharacterized protein LOC577099
uncharacterized protein LOC110984997 isoform X1
endonuclease-reverse transcriptase
craniofacial development protein 2-like
RNA-directed DNA polymerase from mobile element jockey
hypothetical protein DSY43_04445
PREDICTED: uncharacterized protein LOC589347

**"Treated_1 vs Control" and "Treated_1 vs Treated_2"**
annexin A4
heme-binding protein 2
proteasome subunit beta type-1-B
PREDICTED: uncharacterized protein LOC105444208

**"Treated_2 vs Control" and "Treated_1 vs Treated_2"**
PREDICTED: uncharacterized protein LOC105443609
uncharacterized protein LOC110987973
haloacid dehalogenase-like hydrolase domain-containing protein 2
uncharacterized protein LOC111085826
biogenesis of lysosome-related organelles complex 1 subunit 1
PREDICTED: uncharacterized protein LOC105441676
PREDICTED: mucin-4
PREDICTED: uncharacterized protein LOC105437795
PREDICTED: uncharacterized protein LOC105912973
PREDICTED: uncharacterized protein LOC105447150
PREDICTED: uncharacterized protein LOC105441670
protein KRI1 homolog
homeobox protein cut-like 1 isoform X1
low-density lipoprotein receptor-related protein 1B isoform X1
uncharacterized protein LOC110984676 isoform X7
PREDICTED: uncharacterized protein LOC107350581
putative DNA transposase THAP9-like
PREDICTED: uncharacterized protein LOC105437731
PREDICTED: uncharacterized protein LOC754161

PREDICTED: uncharacterized protein LOC578465
PREDICTED: LOW QUALITY PROTEIN: uncharacterized protein LOC107348641
toll-like receptor 3
uncharacterized protein LOC110975147 isoform X1
hypothetical protein AC249_AIPGENE18192
hypothetical protein CIL05_21475
PREDICTED: uncharacterized protein LOC100890507
PREDICTED: uncharacterized protein LOC107334619
long-chain-fatty-acid--CoA ligase 5-like
PREDICTED: uncharacterized protein LOC100893480 isoform X5
hypothetical protein AWC38_SpisGene22463
PREDICTED: sialin
PREDICTED: plexin-B3-like
hypothetical protein BSL78_27382
PREDICTED: uncharacterized protein LOC105440726
PREDICTED: uncharacterized protein LOC105437364
PREDICTED: uncharacterized protein LOC105442164, partial
uncharacterized protein LOC110234800
pre-rRNA-processing protein TSR1 homolog
PREDICTED: uncharacterized protein LOC105444020
sodium/myo-inositol cotransporter 2 isoform X1
PREDICTED: mucin-22-like
deleted in malignant brain tumors 1 protein-like
cystine/glutamate transporter
V(D)J recombination-activating protein 1-like isoform X2
zinc-carboxypeptidase, putative
PREDICTED: uncharacterized protein LOC100890950
PREDICTED: uncharacterized protein LOC108087055
PREDICTED: latrophilin-1
PREDICTED: uncharacterized protein LOC105442991
recombination activating protein 2-like
PREDICTED: uncharacterized protein LOC105445996
PREDICTED: uncharacterized protein LOC105440296
sucrase-isomaltase, intestinal
PREDICTED: uncharacterized protein LOC105447620, partial
PREDICTED: uncharacterized protein LOC105445209
hypothetical protein BSL78_14139
hypothetical protein BSL78_24528, partial
PREDICTED: uncharacterized protein LOC105443558
uncharacterized protein LOC115369174
hyphally regulated cell wall protein 3
PREDICTED: uncharacterized protein LOC100893587
uncharacterized protein LOC110464703

Supplementary Table S13. Common down-regulated genes in the Venn diagrams (for more details see also **Fig. 5** and legend to this figure) among the three experimental groups: "Treated_1 (plutei deriving from adults exposed for two months to sediment contaminated with PAHs) versus Control" (plutei from adults sea urchin *P. lividus* reared for two months in tanks with sediment without contaminants, "Treated_2 (plutei deriving from adults exposed for two months to sediment contaminated with PCBSs) versus Control" and "Treated_1 versus Treated_2".

**Down-regulated genes**
**"Treated_1 vs Control" and "Treated_2 vs Control"**
PREDICTED: uncharacterized protein LOC105440703 isoform X1
E3 ubiquitin-protein ligase TRIM33-like
cGMP-dependent protein kinase 1 isoform X5
PREDICTED: uncharacterized protein LOC105441665
zinc finger protein 862-like
galactosylceramide sulfotransferase-like
UDP-glucuronosyltransferase 2B33-like
PREDICTED: uncharacterized protein LOC105444646
PREDICTED: uncharacterized protein LOC105444794
methylmalonyl-CoA epimerase, mitochondrial
neurogenic locus notch homolog protein 1
zinc finger and SCAN domain-containing protein 29-like
PREDICTED: uncharacterized protein LOC100888828
solute carrier family 28 member 3 isoform X1
uncharacterized protein LOC110061264
PREDICTED: uncharacterized protein LOC109618538
hypothetical protein CRYPA_1785
PREDICTED: uncharacterized protein LOC764539
uncharacterized protein LOC113684170, partial
EGF, latrophilin and seven transmembrane domain-containing protein 1-like
AAEL014742-PA, partial
serine/arginine-rich SC35-like splicing factor SCL33
PREDICTED: uncharacterized protein LOC100893955
fibrillin-1-like
structural maintenance of chromosomes protein 1A isoform X2
uncharacterized protein LOC111113885
gastrula zinc finger protein XlCGF8.2DB-like
PREDICTED: uncharacterized protein LOC105447180
PREDICTED: uncharacterized protein LOC105444769
uncharacterized protein LOC111321513
equilibrative nucleoside transporter 1-like
isocitrate dehydrogenase [NADP] cytoplasmic isoform X2
cilia- and flagella-associated protein 221
putative nuclease HARBI1
hypothetical protein AC249_AIPGENE2135

uncharacterized protein LOC110245901
beta-1,3-galactosyltransferase 1-like
zinc transporter 2
uncharacterized protein LOC110441045
cystine/glutamate transporter
uncharacterized protein LOC110977661
PREDICTED: uncharacterized protein LOC107343991
PREDICTED: uncharacterized protein LOC105446914
unnamed protein product
hypothetical protein, partial
uncharacterized protein LOC115366985, partial
protein NYNRIN-like
PREDICTED: uncharacterized protein LOC100893140
uncharacterized protein LOC110239146
PREDICTED: uncharacterized protein LOC105444695
PREDICTED: uncharacterized protein LOC581299 isoform X10
LINE-1 retrotransposable element ORF2 protein
PREDICTED: uncharacterized protein LOC589347
uncharacterized protein LOC110062710, partial
sushi domain-containing protein 2-like
GRIP and coiled-coil domain-containing protein 2-like
beta-1,4-galactosyltransferase 6-like
PREDICTED: uncharacterized protein LOC105441043
RNA-binding protein 34
kremen protein 2
PREDICTED: uncharacterized protein LOC580499
PREDICTED: uncharacterized protein LOC105440451, partial
uncharacterized protein LOC114531265
beta-1,4-galactosyltransferase 6
PREDICTED: uncharacterized protein LOC100888223
DNA (cytosine-5)-methyltransferase 3A
hypothetical protein BSL78_13033
PREDICTED: uncharacterized protein LOC105438475
hypothetical protein DSY43_04445
PREDICTED: uncharacterized protein LOC105437955
PREDICTED: uncharacterized protein LOC105439418
heparan sulfate glucosamine 3-O-sulfotransferase 1-like
DENN domain-containing protein 4C
Exodeoxyribonuclease III
PREDICTED: uncharacterized protein LOC105440670
PREDICTED: uncharacterized protein LOC588798, partial
PREDICTED: uncharacterized protein LOC105443839
PREDICTED: chondroadherin-like

PREDICTED: uncharacterized protein LOC100890838
centrosome-associated protein CEP250 isoform X4
uncharacterized protein LOC115383145
PREDICTED: uncharacterized protein LOC105444738
tripartite motif-containing protein 5-like
rho-related BTB domain-containing protein 1
prostatic spermine-binding protein-like
probable thiopurine S-methyltransferase
PREDICTED: uncharacterized protein LOC105439906
hypothetical protein LOTGIDRAFT_145246, partial
acid ceramidase
hypothetical protein DJ031_00320, partial
lysine-specific demethylase 8
Transposon TX1 uncharacterized 149 kDa protein
hydroxyacid oxidase 1
uncharacterized protein LOC114967315
deleted in malignant brain tumors 1 protein
hypothetical protein KP79_PYT01408
zinc transporter ZIP10
piggyBac transposable element-derived protein 4-like
PREDICTED: uncharacterized protein LOC105339229
phospholipase B1, membrane-associated
4-hydroxyphenylpyruvate dioxygenase-like protein
PREDICTED: uncharacterized protein LOC100368444
transient receptor potential cation channel subfamily A member 1 homolog
PREDICTED: uncharacterized protein LOC764243 isoform X1

"Treated_1 vs Control", "Treated_2 vs Control" and "Treated_1 vs Treated_2"
RNA-directed DNA polymerase from mobile element jockey-like
putative RNA-directed DNA polymerase from transposon BS
reverse transcriptase family protein
hypothetical protein
uncharacterized protein LOC110990766
putative RNA-directed DNA polymerase from transposon X-element
PREDICTED: uncharacterized protein LOC100888345
polyprotein
RNA-directed DNA polymerase from mobile element jockey
P2X purinoceptor 7-like
hypothetical protein DSY43_00385
uncharacterized protein LOC110975082

"Treated_1 vs Control" and "Treated_1 vs Treated_2"
MAM and LDL-receptor class A domain-containing protein 1

PREDICTED: uncharacterized protein LOC105446188
uncharacterized protein LOC106176709
PREDICTED: uncharacterized protein K02A2.6-like

"Treated_2 vs Control" and "Treated_1 vs Treated_2"
putative IQ motif and ankyrin repeat domain-containing protein
PREDICTED: uncharacterized protein LOC584294
PREDICTED: uncharacterized protein LOC105441319
PREDICTED: uncharacterized protein LOC100893260
Extracellular matrix protein 3
PREDICTED: uncharacterized protein LOC105439932 isoform X1
PREDICTED: hyalin
transposase
carboxypeptidase B
serine/threonine-protein phosphatase 6 regulatory ankyrin repeat subunit B-like
GD13022
proteasome subunit beta type-7
PREDICTED: uncharacterized protein LOC590790, partial
transient receptor potential cation channel subfamily A member 1
uncharacterized protein K02A2.6-like
PREDICTED: uncharacterized protein LOC105442928
Retrovirus-related Pol polyprotein from transposon 412
PREDICTED: uncharacterized protein LOC105446133
PREDICTED: uncharacterized protein LOC105437502
mitochondrial 10-formyltetrahydrofolate dehydrogenase isoform X1
PREDICTED: uncharacterized protein LOC106522096
DNA topoisomerase 2-alpha-like
PREDICTED: uncharacterized protein LOC105437506
PREDICTED: uncharacterized protein F54H12.2-like
kinesin-like protein KIF20A
Elongator complex protein 3
carbonyl reductase [NADPH] 1
PREDICTED: uncharacterized protein LOC105437736
non-specific lipid-transfer protein
sulfotransferase 1C2A
low-density lipoprotein receptor-related protein 12-like
histone chaperone ASF1A
hypothetical protein BSL78_19340
sushi, von Willebrand factor type A, EGF and pentraxin domain-containing protein 1
retinol dehydrogenase 8
uncharacterized protein LOC114544649
PREDICTED: uncharacterized protein LOC100891882
uncharacterized protein LOC113008516

PREDICTED: uncharacterized protein LOC100892824
poly(U)-specific endoribonuclease isoform X2
aldehyde dehydrogenase, mitochondrial
PREDICTED: uncharacterized protein LOC100888657 isoform X1
hyaluronidase-1 isoform X2
PREDICTED: uncharacterized protein LOC100890018 isoform X5
gamma-butyrobetaine dioxygenase
microsomal glutathione S-transferase 1
PREDICTED: alpha-amylase
KLF2/4
PREDICTED: uncharacterized protein LOC764164, partial
uncharacterized protein LOC110989726
protein dispatched homolog 1
PREDICTED: uncharacterized protein LOC756169

Supplementary Table S14. Data of expression levels in embryos at the pluteus stage, deriving from sea urchins exposed to PAHs and PCBs, were reported as a fold difference (in red up-expressed genes; in light blue down-expressed genes) from control (represented by embryos deriving from adults of sea urchins reared in tanks with sediment without contaminants) at 48 hpf. Fold differences greater than ± 1.5 were considered significant. The genes were classified according the four functional classes (stress, development/differentiation, skeletogenesis detoxification, reported in the Supplementary Figure S5).

		**PAHs**	**PCBs**
Stress	*ARF1*	-2.1	-0.5
	caspase 3/7	-1.6	0.7
	CASP8	5.0	5.6
	cytb	4.2	5.8
	ERCC3	1.8	0.2
	GRHPR	1.2	0.8
	GS	1.0	5.6
	HIF1A	-0.2	3.4
	hsp56	0.6	6.0
	hsp60	0.4	-0.1
	hsp70	0.1	-0.7
	MTase	3.6	6.9
	NF-kB	0.1	0.1
	PARP	4.6	5.7
	p38 MAPK	0.7	-0.1
	p53	-5.7	-3.8
	SDH	5.7	7.0
	14-3-3 ε	0.3	8.4
Development/Differentiation	*ADMP2*	0.4	0.2
	Alix	2.0	2.8
	Blimp	1.0	2.7
	BP10	0.0	2.4
	BRA	2.8	2.8
	DELTA	0.1	0.2
	δ-2-catenin	-0.7	-0.5
	FOXA	4.3	4.6
	FoxG	1.9	1.0
	Foxo	3.8	3.7
	GFI1	2.5	2.2
	GOOS	7.2	7.9
	hat	-6.4	-5.2
	H3.3	0.0	4.0

	JNK	7.5	8.2
	KIF19	-0.3	7.2
	nodal	-0.1	8.7
	NOTCH	0.0	0.3
	OneCut	4.8	7.9
	SMAD6	0.8	0.5
	sox9	5.2	6.1
	TAK1	7.0	7.5
	tcf4	7.1	7.2
	TCF7	6.0	6.5
	VEGF	-0.5	0.2
	Wnt5	0.8	0.2
	Wnt6	2.9	2.9
	Wnt8	0.1	3.3
Skeletogenesis	*BMP5-7*	0.0	1.2
	C-jun	-0.1	0.0
	Nec	5.1	6.0
	p16	0.9	1.4
	p19	5.2	6.8
	SM30	5.8	13.6
	SM50	10.0	10.6
	uni	-3.6	0.0
Detoxification	*CAT*	0.2	0.5
	MDR1	2.6	2.7
	MT	3.6	3.6
	MT4	2.7	2.0
	MT5	2.3	0.5
	MT6	4.2	4.3
	MT7	2.3	2.6
	MT8	14.7	17.1

Supplementary Table S15. Chemical analyses of heavy metals on sea water collected a time zero (to, corresponding at the beginning of the experiments) and after one week of treatement with zeolite and activated carbon. Data are expressed as µg/L and the standard deviation is also reported for the values that are higher than the reference values.

Heavy metals		t0	1 week
**Al**		< 10	< 10
	SD		
**Sb**		< 2	< 3
	SD		0.6
**As**		24.6	21.5
	SD	5.2	0.7
**Ba**		< 30	90
	SD		16.9
**Be**		< 5	< 5
	SD		
**B**		4179.1	4802
	SD	919.4	771.8
**Cd**		< 5	< 5
	SD		
**Co**		< 2	< 2
	SD		
**Cr**		10	7
	SD	2.3	2.3
**Fe**		< 50	<50
	SD		
**Mn**		2.1	< 1
	SD	0.4	
**Hg**		< 0.5	< 0.5
	SD		
**Mo**		17.7	24.9
	SD	4.1	2
**Ni**		< 10	< 10
	SD		
**Pb**		< 1	< 1
	SD		
**Cu**		< 10	< 10
	SD		
**Se**		99.9	69.5
	SD	22	37.5
**V**		194.3	205
	SD	41	6.4
**Zn**		13.2	13.8
	SD	2.6	0.2

Supplementary Table S16. Chemical analyses of ammonia, nitrates, nitrites an phosphates on sea water collected a time zero (to, corresponding at the beginning of the experiments) and after one week (1 week) of treatement with zeolite and activated carbon. Data are expressed as mg/L and the standard deviation is also reported for the values that are higher than the reference values.

Sample	Ammonia	Nitrite	Nitrate	Phosphates	
					SD
t0	< 0.05	< 0.05	< 1	< 0.15	
1 week	< 0.05	< 0.05	< 1	0.3	0.3

Supplementary Table S17. Sample names, condition and paired reads for each sample. Control: samples deriving from adults reared in tanks with water and sediment without contaminants; Treated 1, samples deriving from adults reared in tanks with sediment contaminated with PAHs; Treated 2, samples deriving from adults reared in tanks with sediment contaminated with PCBs;

Samples	Sample_Name	Condition	#Paired Reads
1	Control_1	Control	21,023,491
2	Control_2		18,633,056
3	Control_3		20,875,354
4	Treated1_1	Treated1	18,430,344
5	Treated1_2		18,277,648
6	Treated1_3		20,716,663
7	Treated2_1	Treated2	20,751,933
8	Treated2_2		19,621,258
9	Treated2_3		20,793,011

Supplementary Table S18. The number of reads obtained for the samples.

Sample	#Reads
**Pool_R1_001**	179,122,758
**Pool_R2_001**	179,122,758

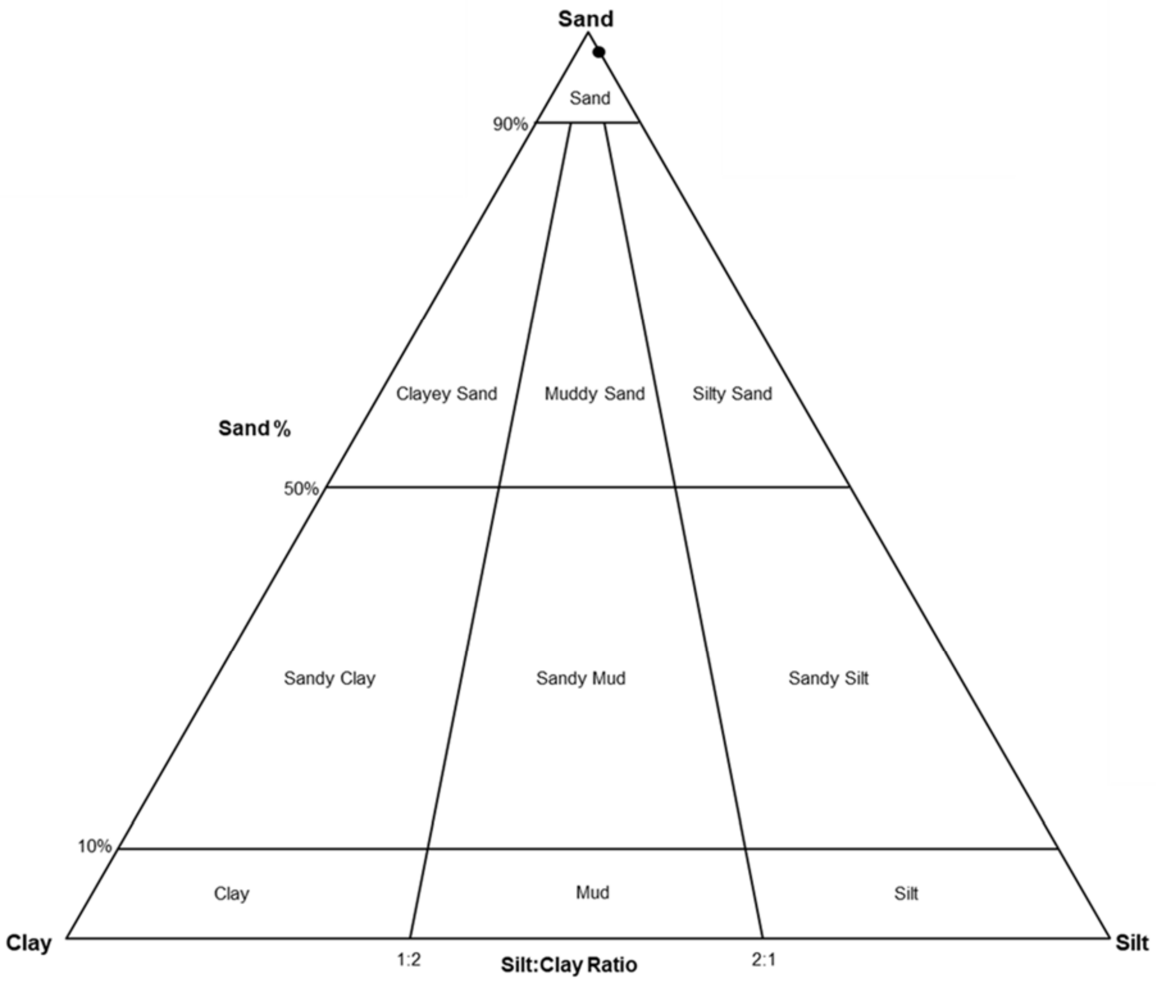

Supplementary Figure S1. Data of the sediment grain size analyzed with GradiStat software (version 8.0 based) to calculate particle size statistics for sieve or laser granulometer data.

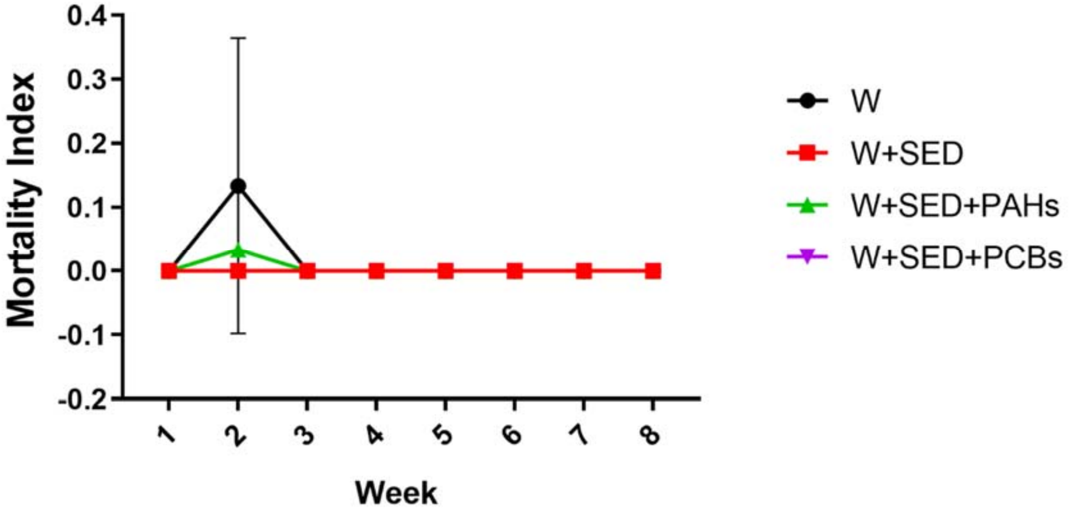

Supplementary Figure S2. Mortality index (calculated as the number of individuals died in a given time period/total number of individuals for the three experimental conditions) of the adult sea urchin P. lividus during the two months of exposure to the sediment contaminated with PAHs at 192 μg/Kg and PCBs 0.15 μg/L and in control condition represented by sediment without contaminants.

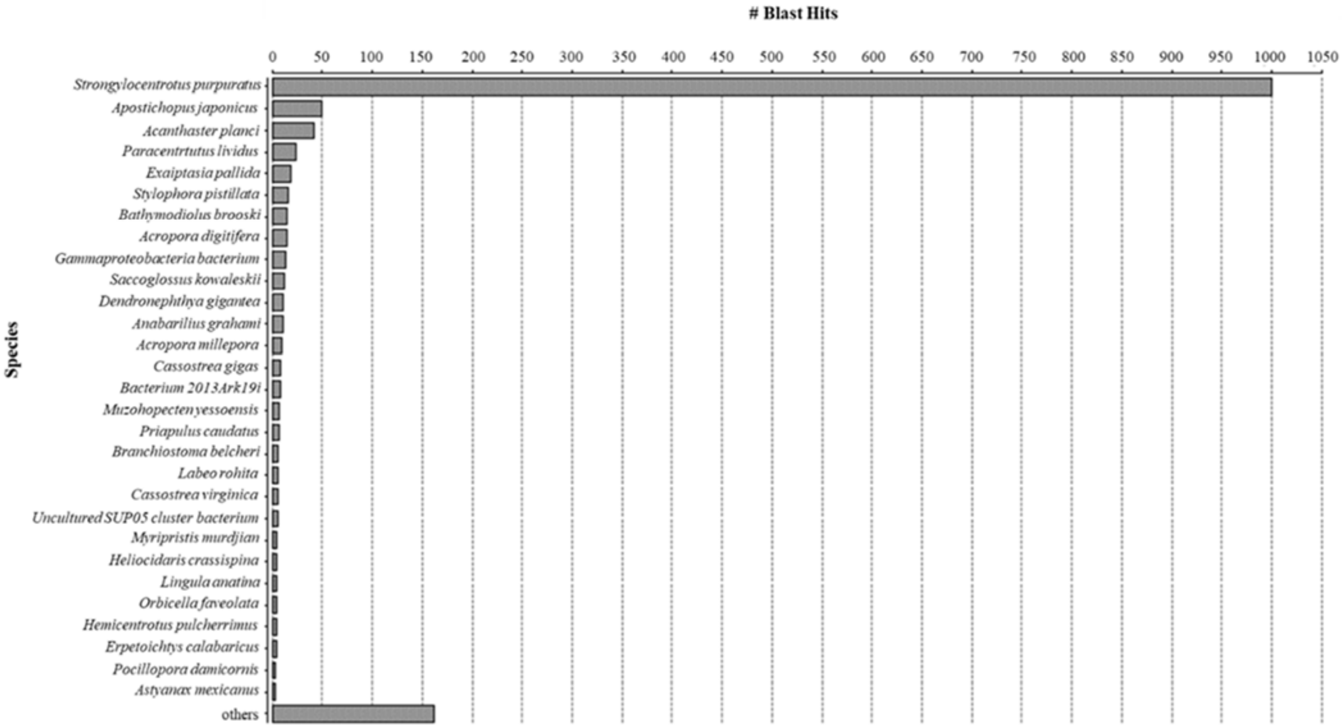

Supplementary Figure S3. Blastx top hit species distribution (reported as number) of matches with known sequences.

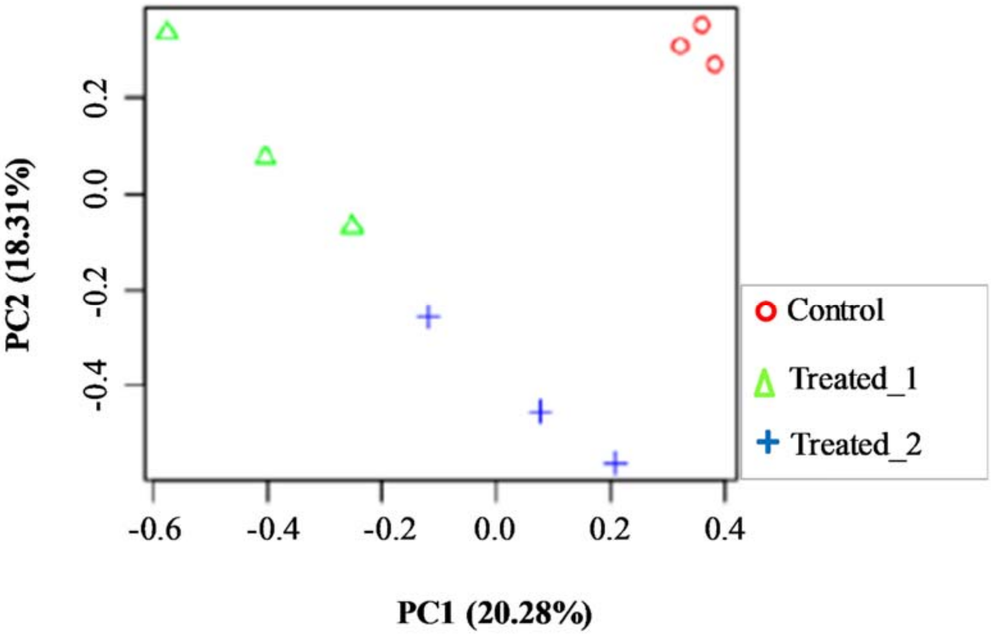

Supplementary Figure S4. Principal Component Analysis (PCA) to evaluate the correlation of samples: Control, Control1, C2 and C3, plutei from adult sea urchin *P. lividus* reared for two months in tanks with sediment without contaminants); the triplicated for plutei deriving from adults exposed for two months to sediment contaminated with PAHs (Treated 1_1, 1_2 and 1_3); the triplicated for plutei deriving from adults exposed for two months to sediment contaminated with PCBs (Treated 2_1, 2_2 and 2_3).

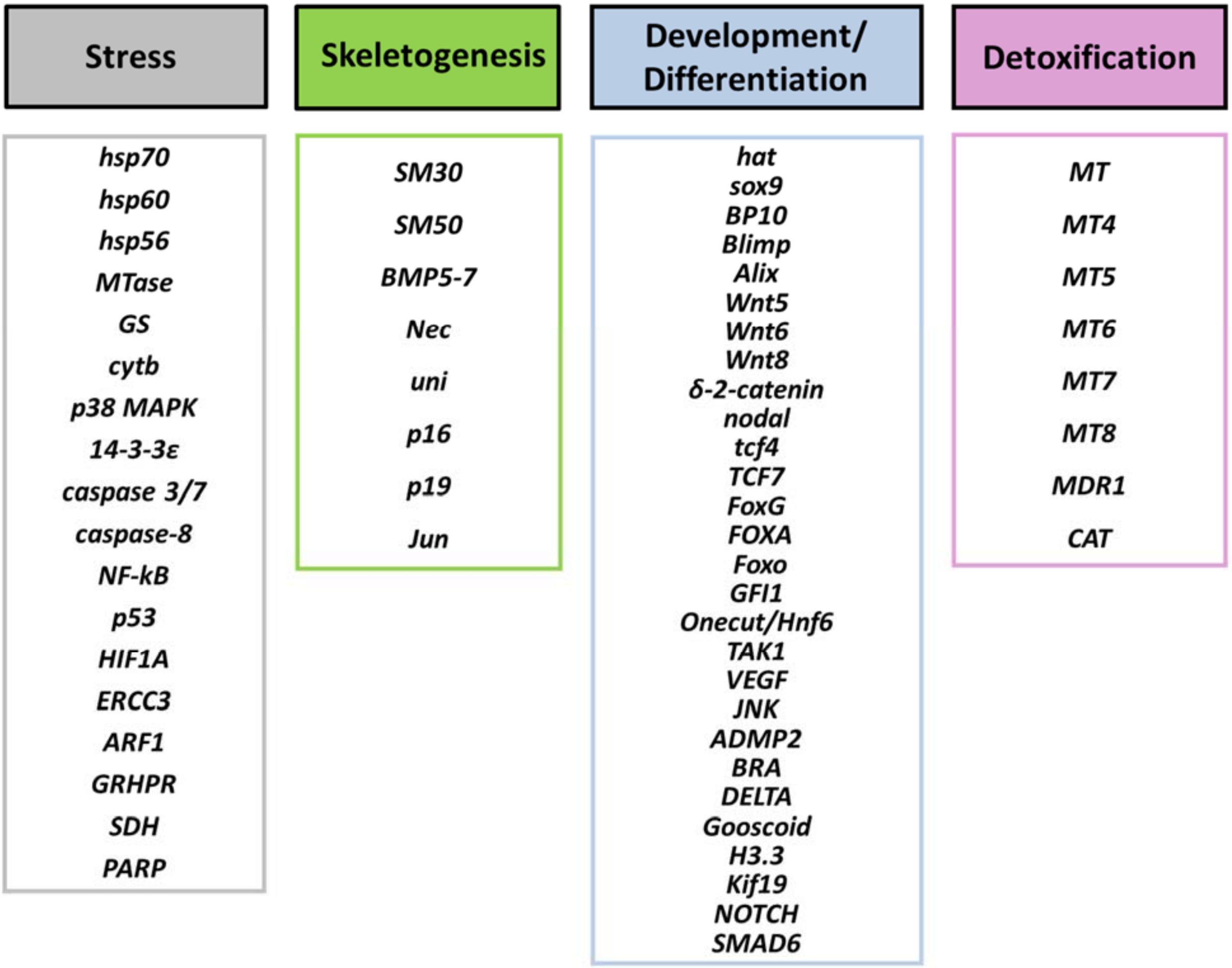

Supplementary Figure S5. Summary of the sixty-two genes analyzed by Real Time qPCR involved in stress, skeletogenensis, development/differentiation and detoxification process. Bibliographic references for the identification of these genes see Varrella et al. (2014), Varrella et al.. (2017), Ruocco et al. (2017), Esposito et al. (2020).

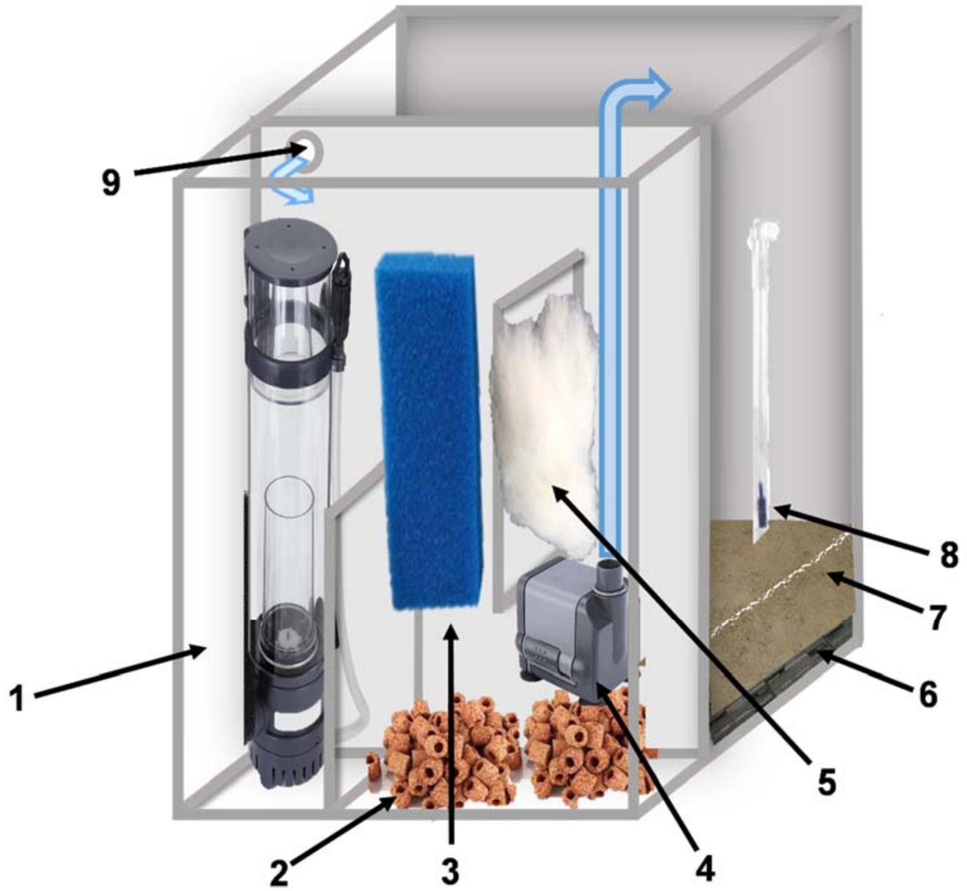

Supplementary Figure S6. Schematic representation (frontal view) of one of the twelve experimental tanks of the mesocosms, with the description of all the elements: (1) protein skimmer to remove organic matter, such as food particles and faecal pellets, using a pump in which establishes a contact between air and water flow, producing fine bubbles by Venturi effect. The water movement promotes the binding of molecules on the bubbles until their interface was saturated. When the bubbles increase on the top of water skimmer, condensed them into a foam collected in the cup. (2) Porous ceramic rings act as biologic filters, favoring the growth of nitrifying bacteria able to oxidize the ammonia into nitrites and then to nitrates. Bacterial activator is also added to promote the growth of *Nitrobacter* and *Nitrosomonas* species that adhere on the porous surface of these filters. (3) Synthetic sponge for a mechanical filtration that together with (5) perlon wool (consisting of thin glass fibres) for trapping particulate matter, such as faecal pellets and sediment suspended particles. (3) Pump for water recirculation. (6) Under-gravel filter to create a constant flow of water through (7) the sediment. (8) Air bubble stone for the aeration and oxygenation the water. (9). Flow of the water from tank to the filter compartment.

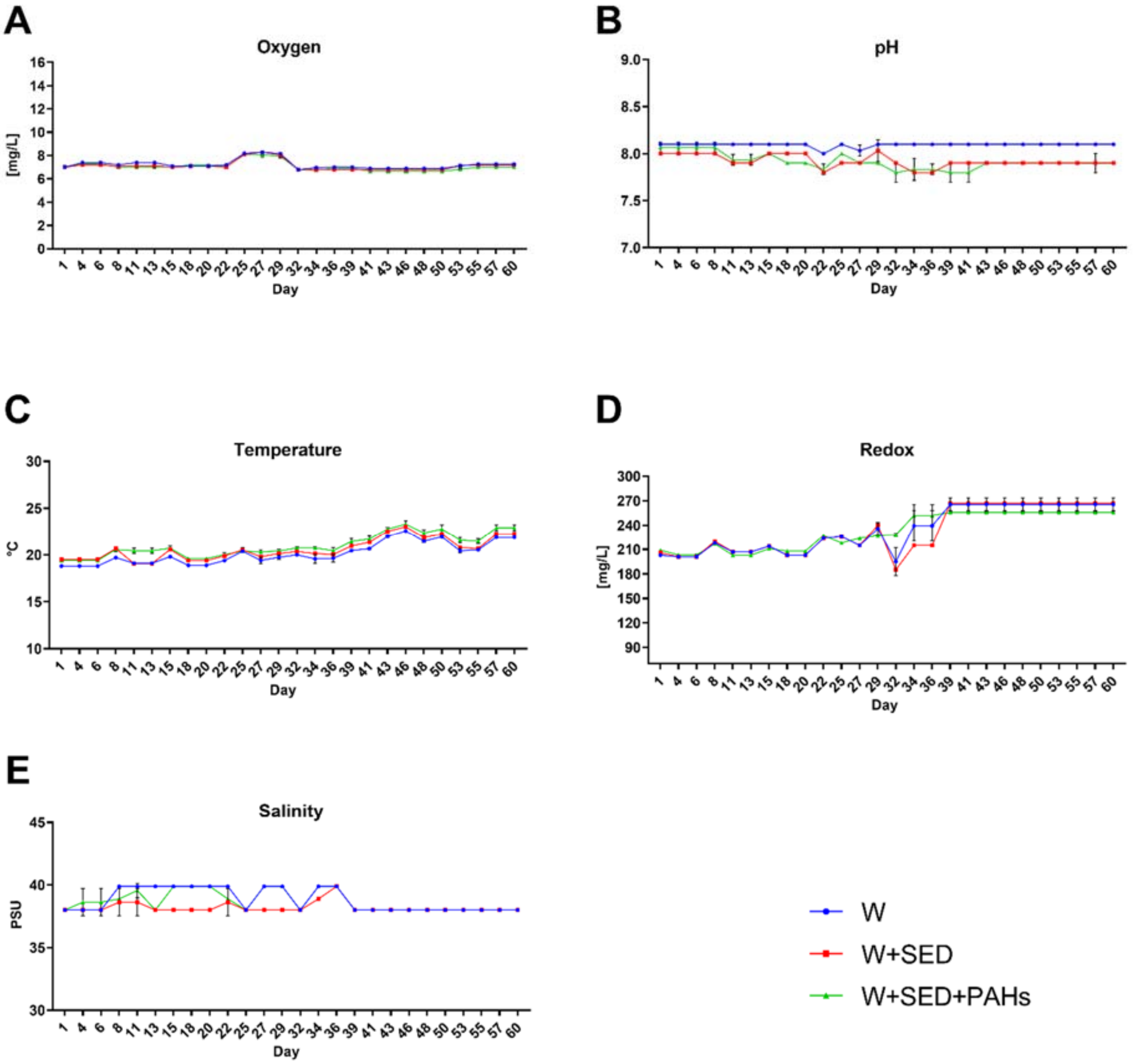

Supplementary Figure S7. Physical parameters (dissolved oxygen, pH,redox, temperature and salinity) of sea water checked three times a week in tanks at the three different experimental conditions: W, W+SED, W+SED+PCBs.

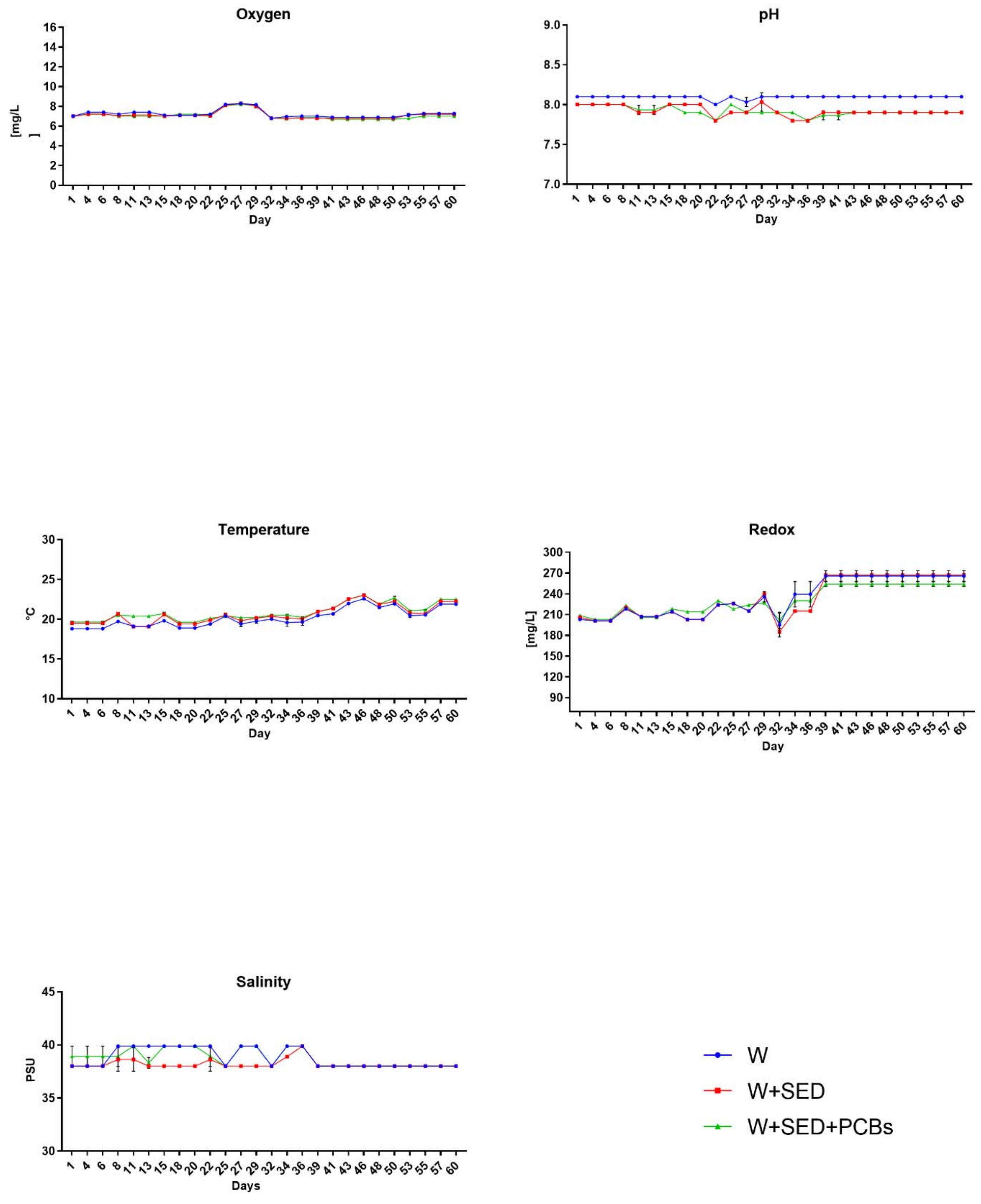

Supplementary Figure S8. Physical parameters (dissolved oxygen, pH, redox, temperature and salinity) of sea water checked three times a week in tanks at the three different experimental conditions: W, W+SED, W+SED+PAHs.

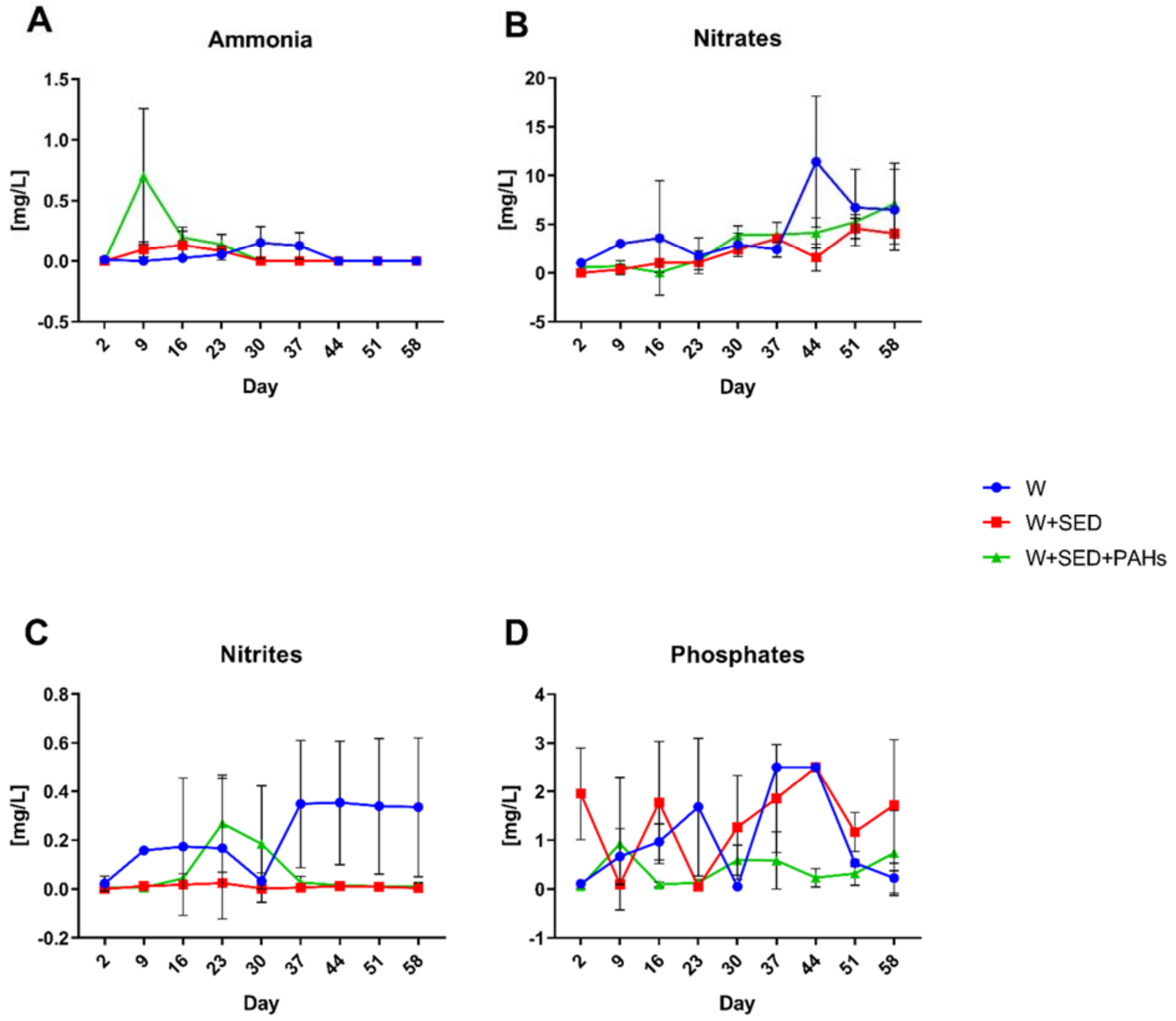

Supplementary Figure S9. Chemical parameters (ammonia, nitrates, nitrites an phosphates) of sea water checked once a week in tanks at the four different experimental conditions: W, W+SED, W+SED+PCBs. On X axis the days at which the data were collected.

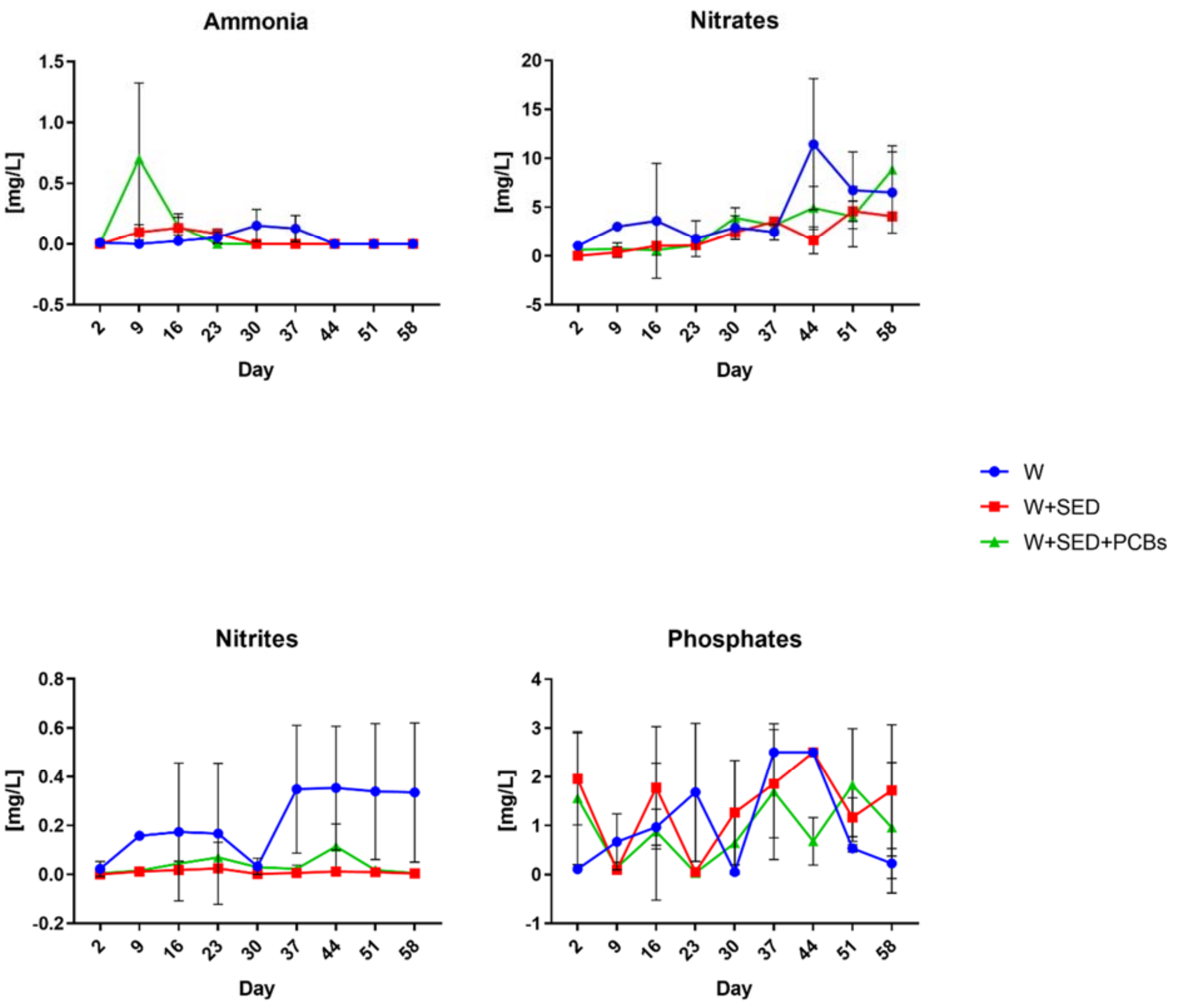

Supplementary Figure S10. Chemical parameters (ammonia, nitrates, nitrites an phosphates) of sea water checked once a week in tanks at the four different experimental conditions: W, W+SED, W+SED+PAHs. On X axis the days at which the data were collected.
